# Supplementary material for: Same-day versus rapid ART initiation in HIV-positive individuals presenting with symptoms of tuberculosis: Protocol for an open-label randomized non-inferiority trial in Lesotho and Malawi
Source: PLoS One. 2024 Feb 8;19(2):e0288944. doi: 10.1371/journal.pone.0288944 (PMC10852279; doi:10.1371/journal.pone.0288944)
Supplement: S1 Checklist — (PDF) [file pone.0288944.s001.pdf]

Clinical Study Protocol SaDAPT Trial

“Same-day versus rapid ART initiation in HIV-positive individuals presenting with symptoms of tuberculosis: an open-label randomized non-inferiority trial in Lesotho and Blantyre district, Malawi”

SaDAPT Trial

**Version Number**	1.3	**Document Date**	14.02.2023
**Study Registration**	Clinicaltrials.gov (NCT05452616)		
**Principal Investigator**	Prof. Dr. Niklaus Labhardt, MD, DTM&H, MIH<br>Head Division of Clinical Epidemiology<br>University Hospital Basel<br>Totengaesslein 3, 4051 Basel, Switzerland		
**Funding Agency**	Swiss National Science Foundation SNSF		

1 GENERAL INFORMATION

I. List of Investigators and other persons involved

Title	Names	Institution	Position	Function in study
Prof. Dr. DTM&H, MIH	Labhardt Niklaus	University of Basel, University Hospital Basel	Head Division of Clinical Epidemiology	Sponsor-Investigator
BMBCh, MSc, DTM&H	Burke Rachael Mary	London School of Hygiene and Tropical Medicine	Research fellow, Deputy Group Head.	Co-Principal Investigator (Co-PI)
Prof. MBBS, PhD	MacPherson Peter	School of Health and Wellbeing, University of Glasgow	Group Leader	Co-Investigator Malawi
MBBS, PhD	Nliwasa Mariott	Helse Nord Tuberculosis Initiative	Group Leader	National PI Malawi
MD PhD	Irene Ayakaka	SolidarMed Lesotho	Technical Director	National PI Lesotho
PD PhD	Glass Tracy	SwissTPH, University of Basel	Group Leader	Statistician
M Med	Gerber Felix	University Hospital Basel University of Basel	PhD Candidate	Co-Investigator
MD PhD	Amstutz Alain	University of Basel, University hospital Basel	Post-doctoral research collaborator	Co-Investigator
MBBS	Kalua Thoko	Centre for International Health, Education, and Biosecurity, University of Maryland Baltimore	Country director	Co-Investigator Malawi
MBBS	Msosa Takondwa	Helse Nord Tuberculosis Initiative	Research Fellow	Co-Investigator Malawi
MBChB	Nthuseng Bridgett Marake	Ministry of Health, Lesotho	HIV Treatment & Care Officer	Co-Investigator Lesotho
MBChB	Lukau Blaise	SolidarMed Lesotho	Medical Officer	Deputy National PI, trial coordinator, study physician, Lesotho
MBBS MSc	Semphere Robina	Helse Nord Tuberculosis Initiative	Research Fellow	Co-Investigator Malawi
MSc	Lee Tristan	SwissTPH, University of Basel	Data Manager	Data Management

II. Table of contents

1 General Information ... 2
I. List of Investigators and other persons involved ... 2
II. Signatures ... **Error! Bookmark not defined.**
III. Table of contents ... 3
IV. Abbreviations ... 6
V. Synopsis ... 7
2 Background information ... 11
2.1 The HIV-TB syndemic ... 11
2.2 Same-day ART initiation ... 11
2.3 ART initiation in PLHIV with presumptive TB ... 11
3 Objectives and purpose ... 13
3.1 Objectives ... 13
3.1.1 Primary objective ... 13
3.1.2 Secondary objectives ... 13
3.1.3 Exploratory objectives ... 13
3.2 Scientific justification and rationale of study population ... 13
4 Study design ... 14
4.1 Endpoints ... 14
4.1.1 Primary endpoint ... 14
4.1.2 Secondary and safety endpoints ... 14
4.1.3 Exploratory endpoints ... 14
4.2 Measures to minimize bias ... 14
4.2.1 Randomisation ... 14
4.2.2 Blinding ... 14
4.3 Study duration and duration of participant's participation ... 15
4.4 Early termination of the study ... 15
4.4.1 Discontinuation on individual level ... 15
4.4.2 Discontinuation of entire study ... 15
5 Selection of the study participants ... 16
5.1 Study setting ... 16
5.1.1 Lesotho ... 16
5.1.2 Blantyre district, Malawi ... 16
5.2 Recruitment ... 17
5.3 Inclusion and exclusion criteria ... 17
5.3.1 Inclusion criteria ... 17
5.3.2 Exclusion criteria ... 17
6 Study procedures ... 18
6.1 Screening and informed consent (study staff) ... 18
6.2 Randomization (study staff) ... 18
6.3 Baseline assessment (study staff) ... 18
6.4 ART initiation (study staff and routine staff) ... 18
6.4.1 "TB results first" arm ... 18
6.4.2 "ART first" arm ... 19
6.5 Further procedures on enrolment day (routine staff) ... 19
6.6 TB test results review, (routine staff) ... 19
6.6.1 Both arms: ... 19

6.6.2 “TB results first” arm: 19
6.6.3 “ART first” arm: 19
6.7 Routine follow up (routine staff) and SAE/AESI surveillance (study staff) 19
6.8 Study visit, week 26 (week 22 - 30) (routine staff and study staff) 20
6.9 Register review, week 30 (study staff) 20
6.10 Tracing for outcome assessment, weeks 31 - 40 (study staff) 20
6.11 Schedule of events 23
7 Safety Considerations 24
7.1 Safety reporting 24
7.1.1 Definition of AEs 24
7.1.2 Definitions of causality for SAEs are as follows 24
7.1.3 Definition of TB-IRIS 25
7.1.4 Surveillance for SAEs and AESIs 26
7.1.5 Management of AEs 26
7.1.6 Reporting of SAEs 26
7.2 Data Safety and Monitoring Board and Interim Assessment 26
8 Description of data management 28
8.1 Specification of source documents 28
8.2 Data management system 28
8.3 Confidentiality and coding 28
8.4 Retention and destruction of study data and biological samples 29
8.5 Data security, access, archiving and back up 29
9 Statistics 30
9.1 Sample size 30
9.2 Analysis 30
9.2.1 Primary analysis 30
9.2.2 Secondary, subgroup and exploratory analysis 30
10 Duties of the Investigators 32
10.1 Investigator’s confirmation 32
10.2 Project management 32
11 Ethical considerations 34
11.1 Independent Ethics Committee (IEC) 34
11.2 Evaluation of the risk-benefit ratio 34
11.3 Participant information and consent 34
11.4 Registration of clinical trial 35
11.5 Participant confidentiality 35
11.6 Participants requiring particular protection 35
11.7 Damage coverage 35
11.8 Participant compensation 35
12 Quality control and quality assurance: description of measures 36
12.1 Risk management and possible constraints 36
12.1.1 Risks to viability of the study 36
12.1.2 Risks and benefits for participants 36
12.2 Monitoring and Auditing 36
12.3 Translations - Reference language 36
12.4 Storage of biological material and related health data 37
12.5 Project timeline 37
13 Funding 38
14 Dissemination of results and publication policy 38

14.1 Dissemination to scientific community; including lead in publications 38
14.2 Information of community and policy makers 38
15 ART initiation cohort sub-study 39
15.1 Rationale 39
15.2 Objectives 39
15.3 Study design and duration 39
15.4 Study population and rationale 39
15.4.1 Inclusion and exclusion criteria 39
Inclusion criteria 39
Exclusion criteria 40
15.4.2 Estimated sample size 40
15.5 Study procedures 40
15.5.1 Screening and informed consent 40
15.5.2 Baseline assessment 40
15.5.3 ART initiation 41
15.5.4 Follow-up 41
15.6 Safety considerations 41
15.7 Data management 41
15.8 Statistics 41
15.9 Ethical considerations 41
15.9.1 Risk benefit assessment 41
15.9.2 Rationale for inclusion of vulnerable subjects 42
16 References 45

III. Abbreviations

AE	Adverse event
AESI	Adverse event of special interest
AIDS	Acquired immunodeficiency syndrome
ALHIV	Adolescents living with HIV
ART	Antiretroviral therapy
CNS	Central nervous system
COU	Clinical Operations Unit
CPT	Cotrimoxazole preventive therapy
CrAg	Cryptococcal antigen
CRF	Case report form
CRSU	Clinical Research Support Unit
DBS	Dried blood spot
DHMT	District Health Management Team
GCP	Good Clinical Practice
ICF	Informed consent form
HIV	Human immunodeficiency virus
ICH	International Council on Harmonisation of Technical Requirements for Registration of Pharmaceuticals for Human Use
ICMJE	International Committee of Medical Journal Editors
IEC	Independent Ethics Committee
IRIS	Immune reconstitution inflammatory syndrome
ITT	Intention to treat (analysis population)
KUHES	Kamuzu University of Health Sciences
PLHIV	People living with HIV
POC	Point-of-care
PP	Per protocol
RCT	Randomized controlled trial
REDCap	Research Electronic Data Capture
SAE	Serious adverse event
SDI	Same-day initiation
Swiss TPH	Swiss Tropical and Public Health Institute
TB	Tuberculosis
TPT	TB preventive treatment
VL	Viral load
WHO	World health organization
W4SS	WHO four-symptom screening

IV. Synopsis

**Sponsor-Investigator**	Prof. Dr. Niklaus Labhardt, MD, DTM&H, MIH<br>Division of Clinical Epidemiology, University Hospital Basel
**Study Title:**	Same-day versus rapid ART initiation in HIV-positive individuals presenting with symptoms of tuberculosis: an open-label, randomized, controlled clinical trial in Lesotho and Blantyre district, Malawi
**Short Title / Study ID:**	SaDAPT Trial ("Same-Day ART initiation in Presumptive TB")
**Protocol Version and Date:**	Version 1.3, 14.02.2023
**Trial registration:**	Clinicaltrials.gov (NCT05452616)
**Study category and Rationale**	Risk category A. The SaDAPT trial entails the comparison of two different algorithms for the timing of ART initiation in people living with HIV (PLHIV) presenting with symptoms of a possible tuberculosis (TB) infection but no signs of central nervous system (CNS) disease.
**Clinical Phase:**	Therapeutic use trial. The trial uses treatments and drug-doses as per international and national guidelines. All treatment components will be applied at standard dosage and no new substances or alternative indications will be tested.
**Background and Rationale**	HIV remains a major cause of morbidity and premature death in many sub-Saharan African countries including Lesotho and Malawi. Globally, 680'000 lives were lost in 2020 in association with HIV despite the availability of effective and low-cost antiretroviral therapy (ART).[1] The most important opportunistic infection associated with HIV is TB, accounting for over 200'000 HIV-related deaths worldwide, mainly in low-income settings, where prevalence of HIV/TB-coinfection is highest.[2] WHO recommends a four-symptom screening (W4SS) approach including cough, fever, night sweat and weight loss for clinical routine TB-screening among PLHIV.[3–6] PLHIV presenting with at least one of the four symptoms are defined as having presumptive TB. The prevalence of presumptive TB among PLHIV not taking ART has been estimated at 71% in a systematic review.[4]<br><br>An important approach to improve access to ART—and thereby reduce HIV transmission as well as AIDS related morbidity and mortality—is the implementation of rapid, and if possible same-day initiation (SDI) of ART.[7] PLHIV with opportunistic infections may benefit particularly from rapid ART initiation and the subsequent suppression of HIV replication and reconstitution of CD4-cell mediated immunity. At the same time, they are at risk of developing immune reconstitution inflammatory syndrome (IRIS) after initiation of ART.[8–10] The risk for development of IRIS increases with earlier initiation of ART.[8,10–13] Until the release of a guideline update in 2021,[3,14] WHO had recommended to delay initiation of ART in case of presumptive TB until TB has been investigated and TB treatment initiated if TB disease has been confirmed in order to reduce the risk of IRIS.[15] The 2021 guideline update contains for the first time a "clinical consideration" to start ART in PLHIV with presumptive TB but no signs of central nervous system (CNS) disease while rapidly investigating for TB, thus allowing SDI for this subgroup of PLHIV. However, a systematic review on the effect of SDI for PLHIV with presumptive TB but no signs of CNS disease, that was conducted to inform this guideline update came to the conclusion that "there is insufficient evidence about whether presence of TB symptoms should lead to ART start being deferred or not".[16] Accordingly, the guidelines emphasize the need for further research on the impact of SDI in PLHIV with presumptive TB on various health outcomes including mortality, HIV and TB outcomes, retention in care, adverse events and IRIS.[14]

**Overall objective:**	To compare two approaches for the timing of ART initiation in PLHIV with presumptive TB but no signs of CNS disease ("ART first" versus "TB results first") with regard to HIV viral suppression, engagement in care, serious adverse events (SAEs) and adverse events (AEs) consistent with TB-IRIS (AEs of special interest, AESIs) in a pragmatic randomized trial reflecting routine primary and secondary care setting in southern Africa.
**Hypothesis**	For PLHIV with presumptive TB, but no signs of CNS disease, same day ART ("ART first") is non-inferior to rapid ART ("TB results first") for being retained in care with suppressed HIV viral load (VL) 26 weeks after enrolment.
**Endpoints:**	**Primary endpoint**<br>- HIV viral suppression (VL <400 copies/mL) 26 (range 22 – 40) weeks after enrolment<br>**Secondary and safety endpoints**<br>- Retention in care 26 (22 – 30) weeks after enrolment<br>- Engagement in care 26 (22 – 30) weeks after enrolment<br>- Disengagement from care 26 (22 – 30) weeks after enrolment<br>- Lost to follow-up 26 (22 – 30) weeks after enrolment<br>- Non-traumatic mortality, SAEs, and AESIs (see section 7.1 for definition) during the first 30 weeks after enrolment<br>- Incidence of TB disease (microbiologically confirmed and/or clinical diagnosis) during the first 30 weeks after enrolment<br>- HIV viral suppression at 26 (22 – 40) weeks using different thresholds (<20 copies/mL; <100 copies/mL; <1000 copies/mL)<br>- ART initiation within 7 and within 28 days after enrolment
**Study design:**	Prospective, parallel, open-label, 1:1 individually randomized, non-inferiority trial
**Inclusion / Exclusion criteria:**	**Inclusion criteria**<br>- 12 years or older<br>- HIV-positive<br>- Not taking ART (naïve or reported no ART intake since 90 days or more)<br>- Unknown TB status<br>- Presenting with one or more TB symptoms according to W4SS[4]<br>- Planning to continue care at the study facility for at least 30 weeks<br>- Willing and able to consent (age 18 years or older) or assent with guardian consent (age 12 to 17 years)<br>**Exclusion criteria**<br>- Medical condition requiring admission or referral to a higher level health facility at enrolment<br>- Symptoms or clinical signs suggestive for diseases of the CNS<br>- Positive cryptococcal antigen test (CrAg)<br>- Reporting to be pregnant<br>- Taking TB treatment,TB preventive therapy (TPT) or treatment against cryptococcal meningitis

**Measurements and procedures:**	SaDAPT is a pragmatic trial attempting to reflect routine care in primary and secondary health facilities in Blantyre district and Lesotho. Study staff will not interfere in most aspects of patient management but primarily have a documentary role.<br>**Screening and Enrolment**<br>- HIV status documentation<br>- Clinical TB screening using the W4SS[4]<br>- Clinical screening for signs of CNS disease<br>- CrAg test if CD4 cell count is < 200 cells/mm^3 or upon clinical suspicion<br>- TB work-up according to local routine (i.e. Xpert MTB/RIF (Ultra), clinical assessment, chest x-ray)<br>**Randomization**<br>- 1:1 randomization stratified by country<br>**Baseline**<br>- Sociodemographic information<br>- Clinical assessment including WHO clinical HIV staging<br>- Collection of plasma samples<br>**Register review, day 28**<br>- Review routine register and update trial data base to ensure correct and complete documentation of procedures and events since enrolment, especially of ART initiation, TB investigations, initiation of TB treatment or TPT and possible AESIs and SAEs<br>**Routine ART care and SAE/AESI surveillance**<br>- (Self-) referral of participants to the study facility in case of medical complaints during the whole follow-up period<br>- Clinical management by routine staff with referral to study staff for assessment and documentation in case of possible SAE or AESI<br>- Review of relevant routine clinical registers for the follow-up duration by study staff to detect possible SAEs or AESIs not yet captured in the trial data base<br>- Report from site investigators to study physician or National PI in case of possible SAE or AESI for further assessment and documentation by physician if required<br>- Reporting of SAEs to Sponsor-Investigator and ethics committee<br>- Reporting of possible AESIs to independent clinical expert committee for classification<br>**Study visit, week 26 (22-30)**<br>- Study visit scheduled together with routine 6-month ART refill visit<br>- Routine ART care including VL measurement by routine clinical staff<br>- Study assessment including assessment of ART and TB status, and inquiry about SAEs since enrollment<br>**Register review, week 30**<br>- Ensure complete documentation of relevant routine register data since enrolment<br>- Identify participants without a documented VL between week 22 and 30<br>**Tracing for outcome assessment, week 31-40**<br>- Participants without a documented VL between week 22 and 30 will be traced via phone calls and home visits after week 30 to ascertain their outcome
**Study Intervention:**	**"ART first" arm:**<br>- ART initiation on the day of enrolment independent of TB investigations<br>**"TB results first" arm**:<br>- ART initiation only after active TB has been refuted or confirmed
**Number of Participants with Rationale:**	Based on the assumptions of non-inferiority of the primary outcome between the two arms, proportions of viral suppression at 26 weeks of 75% in both arms and selection of a non-inferiority margin of 10%, a power of 80%, and a one-sided alpha level of 0.025, a sample size of 590 participants will be required.

**Study Duration:**	First participant in: October 2022. Expected recruitment period to reach sample size: 12 months. Follow-up duration: maximum 40 weeks. Last participant out: July 2024.
**Study Centre(s):**	Seven primary or secondary health care facilities in Lesotho and four primary health care facilities in Blantyre district, Malawi.
**Statistical Considerations:**	We will perform a non-inferiority comparison between the arms on the primary outcome of viral suppression at 26 weeks. Under the assumptions of the sample size calculation, the proportion of viral suppression in the "TB results first" arm is expected to be 75% and the non-inferiority margin is set at 10%. For analysis, we will use a confidence interval approach. We will do the comparison using both a per-protocol (PP) and a modified intent-to-treat (mITT) population. If the lower bound of the 95% confidence interval for the risk difference excludes -10% in both the PP and mITT population, then the intervention will be considered non-inferior. If the "ART first" arm is found to be non-inferior to the "TB results first" arm, then we will assess for superiority using the mITT set.
**GCP Statement:**	This study will be conducted in compliance with the protocol, the current version of the Declaration of Helsinki, the ICH-GCP as well as all national legal and regulatory requirements.

2 BACKGROUND INFORMATION

2.1 The HIV-TB syndemic

Global efforts have led to a substantial decrease in HIV transmission and mortality over the past decades. Nevertheless, HIV remains a major cause of morbidity and premature death in many sub-Saharan African countries including Lesotho and Malawi, the hosting countries of this trial. 680'000 lives were lost in 2020 in association with an HIV infection, 460'000 of them in sub-Saharan Africa, despite the availability of effective and low-cost antiretroviral therapy (ART).[17]

The most important opportunistic infection associated with HIV is tuberculosis (TB). TB is the deadliest infectious disease globally with an estimated 1.3 million deaths among HIV-negative people and an additional 214'000 among people living with HIV (PLHIV) making it the most important cause of death in PLHIV. In low-income settings, where prevalence of HIV/TB-coinfection is highest, TB has a particularly high burden among PLHIV. Africa bears approximately one third of all deaths caused by HIV-associated TB; of the 214'000 TB deaths among PLHIV 170'000 occur in Africa.[2]

Globally, Lesotho and Malawi, the hosting countries of this study, are among the countries with the highest adult HIV prevalence (21.1% and 8.1% respectively)[17] and TB incidence (650 and 140 per 100'000 patient-years)[2] leading to a particularly active syndemic of the two diseases: About half of people diagnosed with active TB are HIV-positive[2] in the two countries and TB causes about a third of HIV-related deaths in Malawi and more than half of HIV-related deaths in Lesotho.[2,17]

2.2 Same-day ART initiation

An important element to improve access to ART is the implementation of rapid, if possible same-day initiation (SDI) of ART in persons found HIV positive. Rapid or same-day ART initiation improves retention in care, viral suppression and survival compared to a delayed start of ART for people with advanced HIV disease.[18–22] In addition to improving individual health outcomes, SDI also reduces the risk of sexual transmission of HIV.[23] The World Health Organization (WHO) as well as guidelines in the US, Europe and southern Africa including Lesotho and Malawi thus recommend rapid, possibly same-day, ART initiation for patients without contraindications, who are ready and willing to start ART immediately.[3,24–29]

Individuals with advanced HIV disease or opportunistic infections may benefit particularly from rapid ART initiation and the subsequent suppression of HIV replication and reconstitution of CD4 cell mediated immunity. At the same time, patients with advanced HIV disease or opportunistic infections, especially TB, are at increased risk of developing immune reconstitution inflammatory syndrome (IRIS) after initiation of ART.[8–10] IRIS is an acute inflammatory condition often presenting with worsening or new onset of symptoms of underlying opportunistic infections, caused by an exaggerated inflammatory response after restoration of immunity through ART. The risk for development of IRIS increases with lower levels of CD4 cells at baseline, the presence of opportunistic infections, especially TB, and with earlier initiation of ART.[8,10–13] IRIS is most deleterious in cases of central nervous system (CNS) involvement in the form of TB or cryptococcal meningitis. Therefore, rapid initiation of ART does not improve overall mortality in patients with CNS infections, but increases incidence of severe adverse events.[30–32] For manifestations of opportunistic infections such as TB outside the CNS, IRIS is less severe often resolving without medical care and optimal timing for initiation of ART remains unclear.[30,33–35]

2.3 ART initiation in PLHIV with presumptive TB

WHO recommends a four-symptom screening approach including cough, fever, night sweat and weight loss for clinical routine TB-screening among PLHIV.[3–6] PLHIV presenting with at least one of the four symptoms are defined as having presumptive TB, and should subsequently undergo microbiological TB investigations to refute or confirm the diagnosis of active TB. Routine TB investigations typically consist of providing the patient two or three sputum bottles with the instruction to return with filled bottles the next day. If the patient returns with the sputum bottles, the samples are sent for analysis using nucleic acid amplification tests (Xpert MTB/RIF (Ultra)). The prevalence of presumptive TB among PLHIV not

taking ART has been estimated at 71% in a systematic review with considerable variation between studies.[4] Studies in Kenya and South Africa have shown that the subsequent bacteriological work-up confirmed the presence of active TB in only 5%-19% of presumptive TB cases.[36–38]

Until the release of a guideline update in 2021,[3,14] WHO had recommended to delay initiation of ART in case of presumptive TB until TB has been investigated and TB treatment initiated if found TB-positive in order to reduce the risk of IRIS.[15] This recommendation to defer ART for everyone with presumptive TB impairs access to same-day ART initiation for a large share of PLHIV. Whereas from a medical perspective delaying ART by a few days until a TB workup has been completed is reasonable, it may cause harm in resource-limited settings where delays between HIV diagnosis and ART initiation are associated with attrition from care, pre-treatment mortality and substantial out-of-pocket costs for patients, especially in remote areas where walking distances or transport costs to clinics are high.[39–41] In these settings, offering same-day ART to PLHIV with presumptive TB while TB work-up is done in parallel may be a valuable strategy to engage and retain more patients in care and thus improve overall clinical outcomes.

The 2021 WHO guidelines update contains for the first time a "clinical consideration" to start ART in PLHIV with presumptive TB but no signs of CNS disease while rapidly investigating for TB, thus allowing SDI for this subgroup of PLHIV. This is representing an important paradigm shift with the potential to reduce the barrier to rapid initiation of ART for a large proportion of PLHIV. However, a systematic review on the effect of SDI for PLHIV with presumptive TB but no signs of CNS disease, that was conducted to inform this guideline update came to the conclusion that "there is insufficient evidence about whether presence of TB symptoms should lead to ART start being deferred or not".[16] Accordingly, the new guidelines emphasize the need for further research on the impact of SDI in PLHIV with presumptive TB on various health outcomes including mortality, HIV and TB outcomes, retention in care, AEs and IRIS.[3]

In this randomized controlled trial (RCT) we will compare two different, guideline-approved algorithms for ART initiation in PLHIV with presumptive TB. In one arm, the updated WHO approach with SDI for all PLHIV with presumptive TB independent of TB diagnostic work-up will be applied ("ART first"). In the other arm, the algorithm recommended by many national guidelines and previous WHO guidelines with deferral of ART initiation until TB is excluded or confirmed and TB treatment initiated will be applied ("TB results first"). The direct comparison of the two approaches in a pragmatic, two-country RCT conducted in a representative high-prevalence setting will provide important evidence on the open question of optimal timing of ART initiation in the large subgroup of PLHIV with presumptive TB.

3 OBJECTIVES AND PURPOSE

3.1 Objectives

The overall objective of the SaDAPT trial is to compare two approaches for the timing of ART initiation in PLHIV with presumptive TB, but no signs of CNS disease in a pragmatic, randomized, therapeutic use trial reflecting a routine primary and secondary care setting in southern Africa. Both timing approaches ("ART first" and "TB results first") are compatible with current national or international guidelines.

3.1.1 Primary objective

- To assess if same-day ART initiation ("ART first") is non-inferior to rapid ART initiation ("TB results first") with regard to HIV viral suppression (VL <400 copies/mL) 26 weeks after enrolment among PLHIV with presumptive TB

3.1.2 Secondary objectives

- To compare in PLHIV with presumptive TB same-day ART initiation ("ART first") versus rapid ART initiation ("TB results first") with regard to:
    - retention in care
    - disengagement from care
    - unsuppressed VL
    - safety outcomes (SAEs, and AESIs)
    - TB incidence
    - proportion of PLHIV with rapid ART initiation (within 7 and within 28 days)

3.1.3 Exploratory objectives

- To describe the prevalence of TB disease at enrolment
- To assess potential sociodemographic and clinical risk factors for adverse outcomes after same-day or rapid ART initiation
- To assess HIV phylogenetics and resistance at enrolment

3.2 Scientific justification and rationale of study population

SaDAPT is a pragmatic trial, conducted in a population representative of those who would receive the interventions in real-life. The setting is the "public health approach" ART program in high HIV prevalence, low-income countries.

We will recruit adults (age 18 years and older) and adolescents (age 12 to 17 years). Adolescents living with HIV (ALHIV) will be included, because they are an important group starting ART, with particularly poor outcomes compared to adults.[42] Information about the optimal timing of ART initiation for ALHIV is important so that specific public health approaches for ART can be made based on evidence rather than extrapolation from adults.

We will not recruit children (age 11 years or younger) as HIV treatment in children and presentation and diagnosis of childhood TB is substantially different to adult and adolescent HIV and TB. Women who are known to be pregnant (based on self-report) will be excluded because delay of ART initiation in pregnancy might lead to adverse effects on the foetus.[3] Individuals who have CNS symptoms will be excluded because same day ART in the presence of CNS opportunistic infections is known to be harmful due to serious IRIS disease.[31,43]

We will not recruit people without capacity to consent, people who are unwell so that they require admission or transfer to a higher level of healthcare at enrolment, or adolescents who present without a guardian.

Measures in place to ensure wellbeing and appropriate consent from all participants, including vulnerable participants are outlined in section 11.3.

4 STUDY DESIGN

SaDAPT is a prospective, parallel, open-label, 1:1 individually randomized, non-inferiority trial.

4.1 Endpoints

4.1.1 Primary endpoint

- HIV viral suppression <400 copies/mL 26 (22 – 40) weeks after enrolment (obtained from routine laboratory reports at study facility, from laboratory reports of referral facility in case of transfer-out, or from dried blood spot (DBS) sample for participants without documented clinic visit but found during home visit tracing)

4.1.2 Secondary and safety endpoints

- Retention in care 26 (22 – 30) weeks after enrolment, defined as a documented ART clinic visit between 22 and 30 weeks after enrolment
- Engagement in care 26 (22 – 30) weeks after enrolment, defined as reporting regular ART intake, irrespective if a documented visit took place between 22 and 30 weeks after enrolment
- Disengagement from care 26 (22 – 30) weeks after enrolment, defined as non-engaged in care but reached through patient tracing
- Lost to follow-up 26 (22 – 30) weeks after enrolment, defined as non-retained in care and not reached through tracing
- Non-traumatic mortality, SAEs, and AESIs (see section 7.1.3 for definition) during the first 30 weeks after enrolment
- Incidence of TB disease (microbiologically confirmed and/or clinical diagnosis) during the first 30 weeks after enrolment, defined as any TB diagnosis after enrolment not classified as prevalent TB at enrolment (see definition below under exploratory endpoints)
- HIV viral suppression at 26 (22 – 40) weeks using different thresholds (<20 copies/mL; <100 copies/mL; <1000 copies/mL)
- ART initiation within 7 and within 28 days after enrolment

4.1.3 Exploratory endpoints

- Clinical characteristics of participants with non-traumatic hospitalizations and deaths in PLHIV with presumptive TB
- Prevalence of active TB diagnosed at enrolment, defined as TB diagnosed clinically or microbiologically through the TB investigations initiated at enrolment and completed up to a maximum of 28 days after enrolment.

4.2 Measures to minimize bias

4.2.1 Randomization

Participants will be randomly allocated to trial arms, and allocation will be made at the time of enrolment.

We will use block randomization with varying block sizes, stratified by country (Malawi and Lesotho). The randomization lists will be generated electronically by a statistician not involved in the trial, and uploaded to the study database.

4.2.2 Blinding

It is not feasible to blind participants and site investigators to arm allocation. However, we do not believe this will influence our aim of conducting a pragmatic trial evaluating the strategies of "ART first" versus "TB results first".

For endpoint assessment, laboratory staff processing viral load samples will be blinded to arm allocation. Endpoints of death or loss to follow-up will be ascertained by site investigators and therefore not

blinded. For the endpoint assessment of AESI incidence, an expert panel will review clinical data, blinded to arm allocation.

4.3 Study duration and duration of participant's participation

First participant was enrolled in October 2022. A recruitment time of about 18 months is expected to be required to reach the calculated sample size. Final study visit is scheduled 26 (range 22 – 30) weeks after enrolment. If after this period the endpoint could not be assessed because the participant did not return to the study facility, a tracing period between week 31 and 40 is scheduled to ascertain the outcome of these participants.

4.4 Early termination of the study

4.4.1 Discontinuation on individual level

Participants can withdraw their consent to participation at any time. Anonymized data until the withdrawal will remain in the database. All personal data will be deleted in case of withdrawal of consent.

Individual participants will only be removed from the study if information later comes to light that they were not eligible for the study at the time of recruitment (i.e. someone subsequently discovered to have already been taking ART at the time of study enrolment). Female participants falling pregnant during the follow-up period of the study will not be discontinued.

4.4.2 Discontinuation of entire study

As this trial uses licensed drugs and licensed doses for licensed indications in two different timing approaches, the risk of significant harm is low. Therefore, we do not plan to have a formal interim analysis. However, there will be a data safety monitoring board (DSMB) session after 9 months of recruitment to review blinded data regarding safety parameters (see section 0).

Additionally, the Sponsor-Investigator in consultation with Co-Investigators may choose to either discontinue the study, or convene an ad hoc meeting of the DSMB to review unblinded data and to assist decision making about trial continuation at any time. Similarly, if the DSMB has concerns after seeing blinded data, they may ask for a meeting to review unblinded data.

The reasons to discontinue the study, or convene the DSMB to review data and consider discontinuing the study including the following;

- Slow or insufficient recruitment
- Alteration in accepted clinical practice that make the continuation of the study unwise.
- Insufficient support/ willingness of routine staff at the study sites to adhere to the study procedures
- Any other ethical concerns

The Sponsor-Investigator may terminate either the entire study or a single study site.

The Sponsor-Investigator would provide the National PI and the study site(s) concerned written notice submitted at a reasonable time in advance of the intended discontinuation. If the Sponsor-Investigator chooses to terminate or pause the study for safety reasons, he will immediately notify all concerned investigators and subsequently provide written instructions for study termination. The National PIs may also terminate or pause the study at one or several of their sites for a reasonable cause, after providing written notice to the Sponsor-Investigator a reasonable time in advance of the intended discontinuation. In case of discontinuation due to safety concerns, no advance notice is required.

5 SELECTION OF THE STUDY PARTICIPANTS

5.1 Study setting

The study will be conducted at primary and secondary level clinics in Lesotho and Blantyre district, Malawi.

5.1.1 Lesotho

Lesotho is a small, landlocked country surrounded by South Africa. It has the second highest HIV prevalence globally with an adult prevalence of 21.1%.[17] For the trial we plan to recruit participants at seven health facilities in the districts of Butha-Buthe (Butha-Buthe Hospital and Seboche Hospital), Mokhotlong (Mapholaneng Health Centre, Mokhotlong Hospital), Leribe (Motebang Hospital), Berea (Berea Hospital) and Maseru (Senkatana Center of Excellence). Apart from Berea Hospital, we have already collaborated with all sites in previous or ongoing trials.[44] Each of these clinics serves between 1000 and 8000 patients taking ART and (re)engages between 100 and 300 patients on ART per year. In these clinics, direct patient care is usually provided by nurses.

5.1.2 Blantyre district, Malawi

In Malawi, we will recruit participants from four trial sites in the Blantyre district. The HIV and TB prevalence in Blantyre is estimated at 16.7%[45] and 0.9%[46], respectively. The four sites, Bangwe Health Centre, Limbe Health Centre, Zingwangwa Health Centre and Ndirande Health Centre are staffed by mixture of clinical officers (clinicians with three years training), nurses, senior health surveillance assistants and medical assistants (lower cadres of staff). All sites provide HIV testing and care, and TB diagnosis and care services. Bangwe Health Centre in the East of Blantyre, was the site of the PROSPECT trial.[47] We have an existing research office co-located at Bangwe Health Centre and many experienced trial research staff familiar with this location. There are 360 to 480 people who newly initiate ART each year at Bangwe. Ndirande clinic is in the North of Blantyre and was the site of the ACT-TB trial from 2018 to 2020[48], which was a trial of antibiotics as a test-of-treatment for people with TB symptoms whilst awaiting TB tests. They have 300 to 360 people newly initiating ART each year, excluding pregnant women. Limbe clinic is East of Blantyre and was also a site for ACT3 TB trial.[48] There are 420 to 480 people who newly initiate ART each year at Limbe, excluding pregnant women.

5.2 Recruitment

Participants will be recruited among individuals presenting at one of the study clinics. Routine staff of all study clinics will be asked to identify individuals, 12 years or older, testing HIV-positive or known to be HIV-positive and not taking ART to the trial's site investigator. The site investigator will then screen potentially eligible individuals based on the below-mentioned inclusion and exclusion criteria and approach them for consent. See section 6.1 for screening procedures and section 11.3 for information about consent process.

5.3 Inclusion and exclusion criteria

5.3.1 Inclusion criteria

- 12 years or older
- HIV-positive
- Not taking ART (naïve or reported no ART intake since 90 days or more)
- Unknown TB status
- Presenting with one or more TB symptoms according to 4WSS[4]
- Planning to continue care at the study facility for at least 30 weeks
- Consent (age 18 years or older) or assent and guardian consent (age 12 - 17 years)

5.3.2 Exclusion criteria

- Medical condition requiring admission or referral to a higher level health facility at enrolment
- Symptoms or clinical signs suggestive for diseases of the CNS
- Positive cryptococcal antigen test (CrAg)
- Reporting to be pregnant (pregnancy test not required)
- Taking TB treatment,TB preventive therapy (TPT) or treatment against cryptococcal meningitis

6 STUDY PROCEDURES

We are conducting a pragmatic study attempting to reflect routine care with as little influence by the study activities as possible. To facilitate understanding of the flow of events, we are presenting procedures conducted by routine clinic staff and study staff jointly with indication of responsible cadre in the paragraph header. For the content and timing of procedures conducted under the responsibility of routine clinical staff, we are describing what is expected to happen according to local guidelines and practices. However, deviations by routine clinical staff from what is described in the sections below are not considered study protocol deviations and are not affecting the participants' retention in the trial.

6.1 Screening and informed consent (study staff)

See section 11.3 for information about informed consent process, including for vulnerable participants.

Potential participants will be asked for verbal consent to study screening. The screening process including oral screening consent will be documented in the electronic trial database. If screening consent is provided, a site investigator will screen for inclusion and exclusion criteria as outlined in section 5.3 including clinical screening for potential CNS disease. Written consent for study participation will be obtained if all inclusion criteria and no exclusion criteria are met. The study will be explained to individuals identified as potential participants (and their guardian if age 12-17 years) and an information leaflet will be handed out. Consideration will be given to ensuring privacy and confidentiality.

6.2 Randomization (study staff)

Stratified randomization will be performed as outlined in section 4.2.1. Allocation will be revealed to study staff electronically on the study tablet. Study staff will inform routine staff about arm allocation.

6.3 Baseline assessment (study staff)

Enrolment will take place in an environment as private as possible, within the constraints of the clinic setting, so that confidentiality can be assured.

On enrolment, the following procedures will be performed:

- Brief clinical history and physical examination including:
  - HIV, ART, TB and general clinical history
  - Sociodemographic characteristics, health status, possible comorbidities and co-medications
  - Measurement of height and weight with determination of BMI and vital signs
- CD4 cell count
- TB investigations
  - According to local routine practices using Xpert MTB/RIF (Ultra), clinical assessment and chest x-ray if available
  - If a urine lipoarabinomannan (LAM) test or any other additional examinations are performed by the responsible routine clinic staff as part of standard of care, the results will be registered by study staff. No LAM assays will be provided by the study team.
- Collection of two tubes of venous blood for centrifugation and storage and subsequent HIV genotyping and resistance testing or other related research. If venous blood draw is not possible, a dried blood spot (DBS) will be collected.

6.4 ART initiation (study staff and routine staff)

6.4.1 "TB results first" arm

The study staff will instruct routine staff that ART initiation should be deferred until results of TB work-up are available. In case TB is diagnosed on the day of enrolment (positive Xpert MTB/RIF (Ultra), chest

x-ray suggestive of TB, clinical judgement by responsible health care provider or any other TB test (LAM) if part of routine care), TB treatment and ART may be initiated together on that day.

6.4.2 "ART first" arm

The study staff will instruct routine staff to offer same-day ART, independent of completion of TB assessment.

6.5 Further procedures on enrolment day (routine staff)

HIV and ART counselling as well as Cotrimoxazole preventive therapy (CPT) prescription will be done by routine clinic staff, according to national guidelines.[28,29] Depending on the decision of the health care professional in charge and availability at the clinic, further investigations may be conducted, i.e. chest x-ray or urine LAM. The study team will register CPT prescription and results of any additional investigations but not intervene actively.

6.6 TB test results review, (routine staff)

The following procedures are expected to be conducted by routine care providers within one week after enrolment. The study staff will not intervene at this moment.

6.6.1 Both arms:

- Discussion of completed TB work-up
- Diagnosis of TB disease (Xpert MTB/RIF (Ultra) positive, positive chest x-ray and/or clinical judgement by responsible health care professional): start TB treatment as per national guidelines[29,49,50]
- TB disease excluded (Xpert MTB/RIF (Ultra) negative and clinical decision that active TB is unlikely): start TB preventive treatment (TPT) as per national guidelines[28,29]

6.6.2 "TB results first" arm:

- Initiate ART as per national guidelines[28,29]

6.6.3 "ART first" arm:

- Continue ART

6.7 Register review, day 28 (study staff)

Study staff will review routine register entries and compare them to the study database with focus on ART initiation, TB investigations, initiation of TPT or TB treatment and occurrence of SAEs or AESIs. Missing data will be added and erroneous data corrected in the study data base to ensure complete and correct data of the first 28 days after enrolment.

6.8 Routine follow up (routine staff) and SAE/AESI surveillance (study staff)

ART care (including TB symptom screening at each visit) as well as management of medical problems during the follow-up period will be provided by routine staff according to national guidelines[28,29]. Participants will be instructed to return to the study facility in case of medical problems any time during the study follow-up. Visits will be documented by routine clinical staff in the specific routine clinical registers (see section 8.1). The clinical management during study follow-up will remain at the responsibility of the routine clinical staff. Routine clinical staff will be instructed to refer study participants to the study team for further assessment and trial documentation in case of a relevant medical problem, especially if suspecting a possible SAE or AESI. Routine staff will be trained prior to the start of the study to recognize conditions potentially relevant for the study, especially on the recognition of possible SAEs and AESIs. In addition to direct referral of participants by the routine clinical staff, the study team will review all relevant routine registers at the study facility searching all entries made during the follow-up period of an individual participant to screen for documentation of possible SAEs or AESIs not otherwise captured in the trial data base. Study staff may review routine registers any time during

the follow-up period. The periods for which the registers have been reviewed will be documented by the site investigators to avoid double-entries or skipping of time periods. Site investigators must analyze all entries made in the registers during the follow-up period and they must document all possible SAEs and AESIs encountered into the trial data base. Site investigators may document visits without occurrence of a possible SAE or AESI, however documentation of uncomplicated visits is not mandatory.

The study staff's main responsibility during the follow-up period is the assessment and documentation of possible SAEs and AESIs. In case a participant is referred directly to the study staff, the site investigators will perform a brief medical history, a targeted physical examination and document results together with information about diagnostic investigations and prescriptions by routine staff in the trial database. For events notified to the study staff after the participant has left the study clinic or for events detected during the review of routine registers, the site investigators will capture as much information as possible about the event from clinical routine registers, inquiry of responsible routine staff and via phone calls to participants or relatives if required. If site investigators detect a possible SAE or AESI, the study physician or National PI will be informed by the site investigators. The study physician or National PI will assess the data provided by the site investigators and decide whether further assessment and documentation of the case is required. Possible further documentation may be conducted by the study physician or National PI him- or herself or may be delegated to other qualified study staff including the site investigators after reception of instructions for appropriate documentation of the case. All information captured by the study physician or National PI or her/his delegate will be saved in the electronic trial data base together with the information captured previously by the site investigators and will later be used for notification to the Sponsor-Investigator and IECs in case of SAEs or for the assessment by the clinical expert committee for classification in case of possible AESIs.

6.9 Study visit, week 26 (week 22 - 30) (routine staff and study staff)

The study visit will be scheduled together with the routine 6 month ART refill visit. Study staff will ensure that a VL gets measured for the assessment of the study endpoint. In addition to adherence counseling and ART refill by routine staff, study staff will take a targeted clinical history including inquiry about ART and TB status, current health status, medication, adherence and possible SAEs since enrollment. Collection of two tubes of venous blood for centrifugation and storage (or a DBS if venous blood draw is not possible) and subsequent HIV genotyping and resistance testing or other related research (as specified in section 16)

6.10 Register review, week 30 (study staff)

Study staff may review routine registers any time during the follow-up period, however, for all participants a final review 30 weeks after enrolment is mandatory to ensure that the entire follow-up period has been covered and that all relevant routine registers data since enrolment (SAEs, possible AESIs, ART status, VL) are documented in the trial data base. In addition to ensuring complete documentation of relevant routine registry data for the entire follow-up period, site investigators will identify participants without a documented clinic visit during the study visit window (week 22 – 30) who will be traced for outcome ascertainment (see below).

6.11 Tracing for outcome assessment, weeks 31 - 40 (study staff)

Participants without a documented VL measurement at the study facility between week 22 and 30 after enrolment will be traced via phone calls and home visits if required between weeks 31 and 40 after enrolment to ascertain their outcome.

In case of documented transfer out, the referral facility will be contacted to obtain a VL laboratory report and the participants will be contacted via phone for assessment of engagement in care and possible SAEs during the follow-up period.

For participants without a documented VL between week 22 and 30 and no documented transfer out, a DBS and if possible two tubes of venous blood (may not be possible for example for household visits) for VL measurement and storage will be collected and the reasons for not having attended a clinic visit between week 22 and 30 will be inquired together with assessment of engagement in care and possible SAEs during the follow-up period. If participants are not engaged in care anymore, the study team will re-link them by giving them an appointment at the health facility.

In case participants cannot be traced back, but relatives or friends of the participants can be contacted (contacts provided by the participants at enrolment), they will be asked about the presumed outcome of the participant. If friends or relatives report a transfer out, the reported referral facility will be contacted to obtain a VL laboratory report. If a participant has died, circumstances of death (in particular whether death was due to trauma and whether TB was diagnosed prior to death) will be recorded.

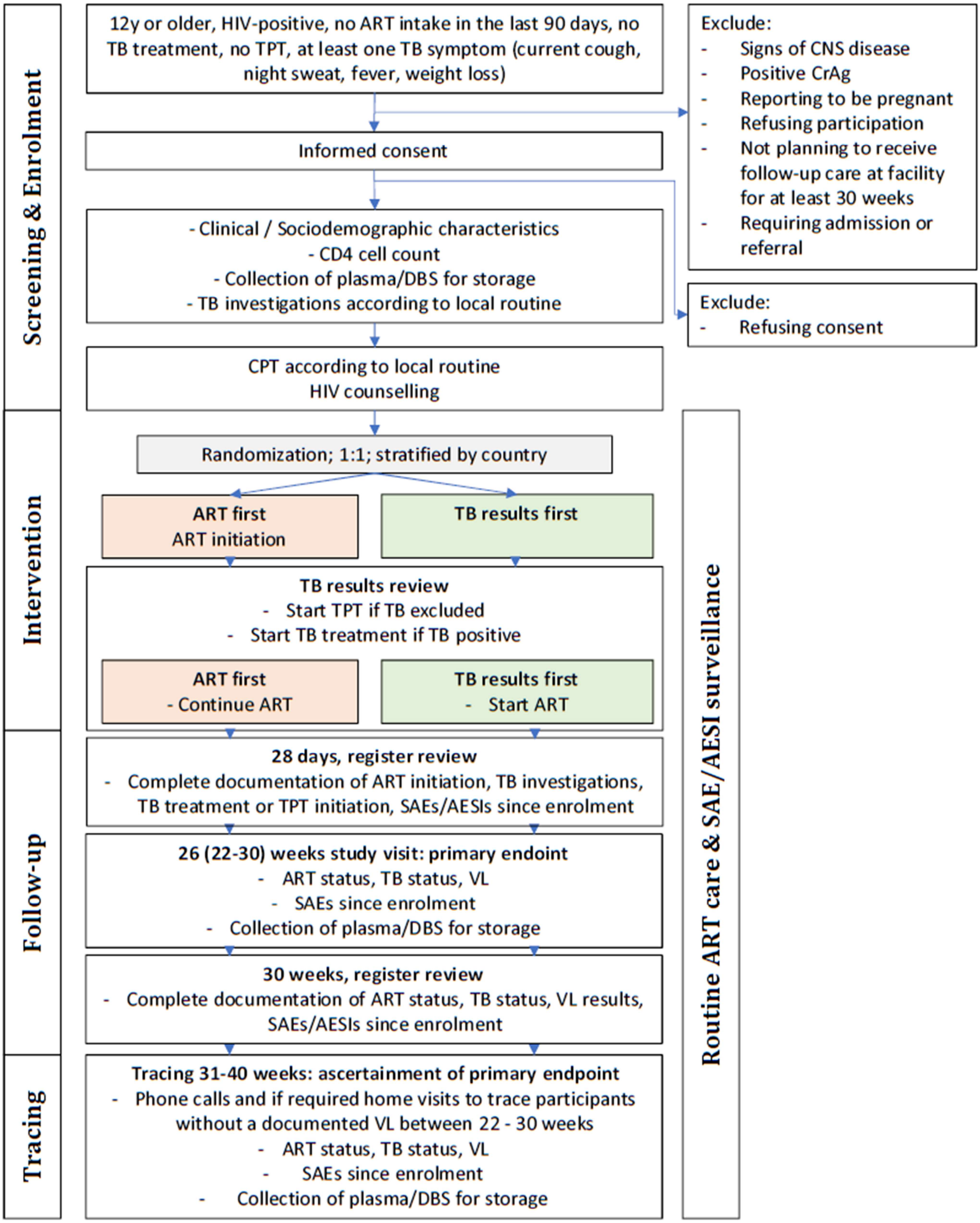

Figure 1 Flow of events SaDAPT Trial *CNS: Central nervous system, CrAg: Cryptococcal antigen, DBS: Dried blood spot, CPT: Cotrimoxazole preventive therapy, TPT: TB preventive therapy, SAE: Serious adverse event, AESI: Adverse event of special interest, VL: Viral load*

6.12 Schedule of events

TIMEPOINT	**0** Screening enrolment	TB results	**28 days** Register review	**Week 26 (22-30)** Study visit	**30 week** Register review	**Weeks 31-40** Tracing[1]
Eligibility screening	X					
Arm allocation	X					
**INTERVENTION [2]:**						
ART initiation ("ART first arm")	X					
ART initiation ("TB results first arm")		X[3]				
**ASSESSMENTS:**						
Questionnaire (including brief medical history)	X			X		(X)
Brief physical examination	X			X		(X)
CD4 cell count	X					
CrAg test if CD4 <200 cells/ml or clinical indication	X					
TB investigations[4]	X					
Collection of plasma samples[5]	X			X		(X)
SAE and AESI surveillance [6]	(X)	(X)		(X)		
HIV Viral Load				X		(X)
Register review: Ensure complete and correct trial data by checking routine registers			X		X	
**ROUTINE CARE:**						
ART counselling	X	X		X		
ART refills [7]				X		(X)
Start TB treatment or TPT [7]		X				
Ongoing clinical care as required	(X)	(X)		(X)		(X)
Start CPT [7]	(X)					

[1]Tracing via phone calls and home visits if no documented VL in endpoint assessment window at 22-30 weeks

[2] ART provision by routine services, the trial intervention is to allocate the timing of ART

[3] ART start if not diagnosed with TB disease, if TB disease diagnosed ART may be delayed up to 14 days– according to local routine

[4] According to local routine

[5] Or DBS if venous blood draw is not possible

[6] AE surveillance; participants, family members and routine staff are asked to contact study team in case of medical complaints. Referral to study team for in case of suspected SAE or TB-IRIS

[7] According to local routine practices

7 SAFETY CONSIDERATIONS

SaDAPT is not a trial of an investigational medicinal product, all drugs used are licensed for the indications and doses used in this trial. Both arms are strategies that have been endorsed by national or international guidelines. SaDAPT is a pragmatic trial embedded within a "public health" ART system, designed to evaluate effectiveness of ART timing strategies in conditions as close to routine practice as possible.

The expected relative safety of the interventions and the desire to alter standard care as little as possible have informed our safety procedures and reporting.

7.1 Safety reporting

7.1.1 Definition of AEs

Adverse event (AE)	Any untoward medical occurrence in a trial participant, including occurrences that are not necessarily caused by or related to that trial procedures.
Adverse event of special interest (AESI)	AE consistent with TB IRIS (see section 7.1.3 for definition) according to judgement of independent clinical expert committee
Serious adverse event (SAE)	Any AE that:<br>o Results in death<br>o Is life-threatening<br>o Requires hospitalization or prolongation of existing hospitalization<br>o Hospitalizations due to uncomplicated delivery are not considered as SAE<br>o Results in persistent or significant disability or incapacity<br>o Consists of a congenital anomaly or birth defect

7.1.2 Definitions of causality for SAEs are as follows

Unrelated	There is no evidence of any causal relationship
Unlikely related	There is little evidence to suggest that there is a causal relationship (for example, the event did not occur within a reasonable time after administration of the trial procedures). There is another reasonable explanation for the event (for example, the patient's clinical condition or other concomitant treatment).
Possibly related	There is some evidence to suggest a causal relationship (for example, because the event occurs within a reasonable time after administration of the trial procedures). However, the influence of other factors may have contributed to the event (for example, the patient's clinical condition).
Probably related	There is evidence to suggest a causal relationship and the influence of other factors is unlikely.
Definitely related	There is clear evidence to suggest a causal relationship and other possible contributing factors can be ruled out.

7.1.3 Definition of TB-IRIS

We will use the Meintjes et. al. definition of paradoxical and unmasking TB-IRIS.[8]

Paradoxical TB IRIS

(A) Antecedent requirements

Both of the two following requirements must be met:

- Diagnosis of tuberculosis: the tuberculosis diagnosis was made before starting ART and this should fulfil WHO criteria for diagnosis of smear-positive pulmonary tuberculosis, smear-negative pulmonary tuberculosis, or extra-pulmonary tuberculosis
- Initial response to tuberculosis treatment: the patient's condition should have stabilised or improved on appropriate tuberculosis treatment before ART initiation—e.g., cessation of night sweats, fevers, cough, and weight loss. (Note: this does not apply to patients starting ART within 2 weeks of starting tuberculosis treatment since insufficient time may have elapsed for a clinical response to be reported)

(B) Clinical criteria

The onset of tuberculosis-associated IRIS manifestations should be within 3 months of ART initiation, re-initiation, or regimen change because of treatment failure. Of the following, at least one major criterion or two minor clinical criteria are required:

Major criteria

- New or enlarging lymph nodes, cold abscesses, or other focal tissue involvement—e.g., tuberculous arthritis
- New or worsening radiological features of tuberculosis (found by chest radiography, abdominal ultrasonography, CT, or MRI)
- New or worsening CNS tuberculosis (meningitis or focal neurological deficit—e.g., caused by tuberculoma)
- New or worsening serositis (pleural effusion, ascites, or pericardial effusion)

Minor criteria

- New or worsening constitutional symptoms such as fever, night sweats, or weight loss
- New or worsening respiratory symptoms such as cough, dyspnea, or stridor
- New or worsening abdominal pain accompanied by peritonitis, hepatomegaly, splenomegaly, or abdominal adenopathy

(C) Alternative explanations for clinical deterioration must be excluded if possible

- Failure of tuberculosis treatment because of tuberculosis drug resistance
- Poor adherence to tuberculosis treatment
- Another opportunistic infection or neoplasm (it is particularly important to exclude an alternative diagnosis in patients with smear-negative pulmonary tuberculosis and extrapulmonary tuberculosis where the initial tuberculosis diagnosis has not been microbiologically confirmed)
- Drug toxicity or reaction

Unmasking tuberculosis-associated IRIS

Patient is not receiving treatment for tuberculosis when ART is initiated and then presents with active tuberculosis within 3 months of starting ART

AND one of the following criteria must be met:

- Heightened intensity of clinical manifestations, particularly if there is evidence of a marked inflammatory component to the presentation. Examples include tuberculosis lymphadenitis or tuberculosis abscesses with prominent acute inflammatory features, presentation with pulmonary tuberculosis that is complicated by respiratory failure due to adult respiratory distress syndrome, and those who present with a marked systemic inflammatory syndrome related to tuberculosis.
- Once established on tuberculosis treatment, a clinical course that is complicated by a paradoxical reaction

The study team will gather as much information as possible about all possible AESIs. Information will be gathered about TB diagnoses, timing of ART initiation (relative to TB diagnosis and treatment), clinical course (including documentation of relevant diagnostic and therapeutic activities by routine clinical staff), and alternative explanations for clinical symptoms. This information (including judgement of study physician, National PI or delegate) will be presented to an independent expert clinical committee for adjudication about likelihood that symptoms are consistent with TB-IRIS. The adjudication committee will be blinded to arm allocation.

7.1.4 Surveillance for SAEs and AESIs

SAEs are solicited in three ways (see section 6)

- o Active inquiry at 26 (22-30) week study visit
- o Register reviews and referral to study team by routine staff in case of relevant medical complaints
- o Tracing with phone calls and home visits between week 31 and 40 to determine outcome for participants without a documented clinic visit between week 22 and 30

AESIs are captured through register reviews and referral to study team by routine staff in case of relevant medical complaints. We will not inquire about AESIs at the 26 (22-30) weeks study visit or during the tracing because the long recall period will not allow for adequate documentation of AEs other than SAEs.

7.1.5 Management of AEs

All participants are under the care of qualified healthcare professionals through the routine ART and TB services and study staff will not intervene in care.

7.1.6 Reporting of SAEs

Site investigators will document any SAE in dedicated CRFs and inform a study physician or the National PI about any SAE.

Study physicians or the National PIs are responsible for making an initial assessment of likely causality and reporting SAEs to the Sponsor-Investigator.

If an SAE is thought to be possibly, probably or definitely related to the study intervention, or if the study physician or National PI is unsure, this should be reported to the Sponsor-Investigator within 72 hours of becoming aware of the event. All deaths should be reported to the Sponsor-Investigator within 72 hours of being aware, regardless of causality. A follow-up report with more details of circumstances of the SAE will be made within 7 days of being aware and communicated to the Sponsor-Investigator. These reports will be reviewed by the Data Safety and Monitoring Board (DSMB).

All SAEs that are thought to be possibly, probably or definitely related to the study interventions will be reported to the ethics committees within 14 days.

7.2 Data Safety and Monitoring Board (DSMB)

7.2.1 Purpose of DSMB

The purpose of the SaDAPT DSMB is to protect the safety of study participants, to assist and advise the SaDAPT steering committee to protect the integrity, validity and credibility of the trial. The main task of the DSMB is to review SAEs and AESIs. Based on the review of SAEs and AESIs, the DSMB members shall flag concerns regarding safety and give recommendations to the SaDAPT Steering Committee whether to continue, amend or halt the trial due to these concerns. The trial protocol does not foresee any interim analysis or interim review of outcome data.

7.2.2 DSMB Members

Based on the task to qualitatively review SAEs/AESIs for potential safety concerns regarding the trial, the DSMB shall consist of three members with expertise in global HIV/TB management and/or clinical research methodology with one of them being appointed to chair the DSMB. The three DSMB members may ask the SaDAPT steering committee for an additional expert (i.e. statistician) to be appointed if required at any time.

7.2.3 DSMB Procedures

If either the SaDAPT steering committee, the Sponsor-Investigator or any of the DSMB members has safety concerns during the trial, or if the steering committee or Sponsor-Investigator wish to seek advice from DSMB they may request a DSMB meeting to review SAEs/AESIs at any time.

If no party raises concerns or requests an earlier meeting, the DSMB will convene after 15% of the projected total number of participants (total number = 590, 15% = 89) have passed the primary endpoint window (30 weeks after enrolment). Thereafter, meetings will be scheduled 6-monthly until the last participant has completed the trial.

Ahead of the meetings, the DSMB members will receive a report including all SAEs/AESIs reported until then. DSMB members review the report for potential relation of the SAE/AESI with the trial procedures. The DSMB may ask for further data on specific SAEs/AESIs if needed. Based on this assessment the DSMB then issues recommendations regarding continuation, amendment or interruption of the trial.

Further details about the DSMB responsibilities and procedures will be outlined in a separate DSMB charter.

8 Description of data management

8.1 Specification of source documents

Source data includes the original records from which information related to the study is derived and may be in electronic or paper format. The source documents for this study include the completed electronic case report forms (eCRF) in the study database for results of physical examination and medical history that go beyond the detail documented in routine clinical registers, as well as ART registers, pharmacy / dispensing ledgers, TB test registers, TB diagnosis registers and routine clinic electronic medical records systems. Finally, information may be collected from computers in the diagnostic laboratory and paper-based laboratory reports.

8.2 Data management system

A comprehensive Data Management Plan will be developed prior to the start of data collection, which will outline the data management products, software and procedures for data collection, management and review. All study team members responsible for data entry and data management will be appropriately trained. The National Principal Investigators have the overall responsibility for data quality and will take reasonable measures to ensure the completeness and accuracy of the study data. Data managers and monitors will regularly review the data. Queries regarding inconsistencies, incoherencies and missing data will be raised within the electronic data capture (EDC) system. All user-specific data processing operations (creation, modification and deletion of patient records) will be recorded in a computer-generated time stamped audit trail.

Data collection will be conducted online (with offline data collection possible where internet or mobile connectivity is insufficient) via the eCRFs developed in in a validated installation of the Research Electronic Data Capture (REDCap) platform[51], validated by the Department of Clinical Research (DKF) at the University Hospital Basel, Switzerland. REDCap supports online and offline data capture and provides compliance with 21 CFR Part 11, FISMA, HIPAA, and GDPR. Study data related to screening, demographics, medical history, clinical examination findings, laboratory results and tracing information will be collected and entered into the eCRF by dedicated study personnel using password protected tablets. The EDC system will be hosted by the Department of Clinical Research (DKF) at the University Hospital Basel, Switzerland, and stored on a secure and encrypted server maintained by the IT-department of the University Hospital Basel. Data management will be performed by the Clinical Statistics and Data Management Unit, Swiss TPH.

8.3 Confidentiality and coding

At all times, the study data will be handled with the uttermost discretion and only be accessible by authorized personnel who require the data to fulfil their duties within the scope of the project. Each site will have a single, password-protected tablet. The eCRF will contain a master list form capturing personally identifying information such as name, date of birth and contact details for the purpose of identification by site investigators during follow-up, including tracing. Identifiable data will only be accessible by the respective site investigator for study implementation and data collection purposes and the trial data manager overseeing the electronic database, and will not be included in any exported data or metadata. Study participants will be allocated a unique participant number, which will be used to identify participants in the eCRFs and in all data exports. The investigators will respect participants' privacy according to all applicable privacy laws. Only anonymized study data will be published in scientific journals and presented at scientific meetings and conferences. All participant personal and medical information are confidential and disclosure to third parties is prohibited. Access to identifiable data for the purpose of data quality control may be granted upon request to the National Principal Investigator, the Sponsor-Investigator and other members of the study team responsible for data quality control.

8.4 Retention and destruction of study data and biological samples

At the conclusion of the study or premature termination, all study data will be locked and archived. The electronic database will be locked and a complete study dataset will be transferred to the statistician and Sponsor-Investigator through a secure channel. The eCRF and study data will be stored in the EDC software and stored by DKF on the University Hospital Basel servers for 25 years. Paper-based informed consent forms will be kept at the study sites with the site investigators being responsible for safe storage or at a later phase at a central point in each country with the National PI being responsible for safe storage for at least 10 years after termination of the study. Collected DBS and plasma samples testing will be kept for a maximum of five years and destroyed thereafter. In Lesotho, samples will be stored in the Seboche Hospital Laboratory; in Malawi at the Kamuzu University of Health Sciences TB laboratory.

8.5 Data security, access, archiving and back up

All study data will be managed securely, in accordance with local regulations and encrypted and password protected in the EDC system, including a full audit trail. All principles of European General Data Protection Regulations will apply, unless these conflict with Malawi or Lesotho regulations, in which case the national regulations will apply.

Direct access to eCRFs and the digital data base will be permitted for purposes of monitoring, audits and inspections.

The EDC system will allow for a database freeze to avoid data from being changed by data collectors without authorization from the Data Manager. A database lock will also be enabled that will be used for final statistical analyses and prevent any further changes after final data entry and query resolution. This will be implemented according to agreed timelines.

The database design specification, study data and metadata will be archived by the trial data management on Swiss TPH servers for 25 years. For the servers on which study data will be stored, there is a defined policy in place for server set up, maintenance and security. This includes processes regarding server qualification, backups, disaster recovery and restricted server access.

Following completion of the study, anonymized individual data will be made freely available on a suitable repository concurrent with publication of initial results. Participants will be asked to consent to this at the time of enrolment.

Further details will be outlined in the Data Management Plan.

9 STATISTICS

9.1 Sample size

The sample size has been calculated with regard to the primary endpoint of the study. Secondary endpoints have not been considered for the determination of the sample size. The expected proportion with viral suppression at 26 weeks after enrolment in the "TB results first" arm is expected to be 75%. To test the hypothesis of non-inferiority of the primary outcome between the two arms, we set the non-inferiority margin to 10%, the power to 80%, and a one-sided alpha level of 0.025. These assumptions yield a sample size of 295 participants per arm.

9.2 Analysis

All analyses will be done by the trial statistician using R (the R Foundation for Statistical Computing) or Stata (version 14, Stata Corporation, Austin/Texas, USA). Analysis will follow CONSORT guidelines.[52,53] A flowchart will describe the inclusion and follow-up of participants by study arm. Baseline characteristics will be described by study arm with summary statistics such as median and interquartile range or number and percentage; no formal testing between arms will be performed. Outcomes will be described by arm using summary statistics. A detailed statistical analysis plan will be developed separately

The following analysis sets will be used in this trial:

1. Modified Intention-to-treat (mITT): anyone found to be ineligible after randomization (i.e., pregnant, had not been off ART for ≥90 days, etc.) will be excluded.
2. Per-protocol (PP) set: This set includes all participants who completed the study without a major protocol deviation (definitions in Statistical Analysis Plan).
3. Complete-case set: all individuals for which the endpoint data could be ascertained.

9.2.1 Primary analysis

The primary analysis for this study will be the comparison of viral suppression rates between the two study arms. Individuals who do not have documented VL 22 – 40 weeks after enrolment, died, transferred out without a documented VL, or were lost to follow-up will be considered to have a negative outcome (i.e., not achieved viral suppression). The analysis will use a logistic regression model adjusted for the pre-specified randomization stratification factors.[54] Moreover, we will adjust for the most important baseline characteristics if found to be unbalanced (gender, age, ART naive vs reinitiating ART, number and type of TB symptoms) between study arms.[55] For the non-inferiority comparison between the two arms, a CI approach will be used. A figure illustrating the confidence intervals for the risk differences and the non-inferiority margin will be presented. If the lower bound of the confidence interval for the risk difference does not include the non-inferiority margin, then the intervention will be considered non-inferior. Primary analyses for the non-inferiority comparison will be performed on the mITT, per-protocol, and complete case sets.[56] If the "ART first" arm is found to be non-inferior to the "TB results first" arm in the mITT and per-protocol sets, then we will assess for superiority using the mITT set. Further details will be provided in the statistical analysis plan.

As a sensitivity analysis for the primary analysis, primary endpoint information obtained from the tracing visit will not be included.

9.2.2 Secondary, subgroup and exploratory analysis

The secondary endpoints of ART initiation within 7 or 28 days after enrollment and retention in care will be assessed for superiority. Secondary endpoints will be analyzed with logistic regression models adjusted for stratification factors and results will be presented as risk differences and 95% confidence intervals.

Non-traumatic mortality, hospitalizations, SAEs, AESIs and incidence of TB will be summarized descriptively as events are expected to be rare.

We plan to assess the potential effect modification of key factors (age groups, CD4-count, country) on the primary outcome by including interaction terms in the model. If the interaction term is found to be significant, effect estimates will be summarized descriptively by subgroup. The study is not powered for these pre-planned subgroup analyses so all results will be considered exploratory.

The statistical analysis plan will provide all further details.

10 DUTIES OF THE INVESTIGATORS

10.1 Investigator's confirmation

This study will be conducted in compliance with this protocol, the International Conference on Harmonisation Good Clinical Practice E6 (R2) (ICH-GCP) and the current version of the Declaration of Helsinki.

All protocol modifications will be documented in writing. A protocol amendment can be initiated by either the Sponsor-Investigator or any investigator. The concerned investigator will provide the reasons for the proposed amendment in writing and will discuss with the Sponsor-Investigator. Any protocol amendment must be approved and signed by the Sponsor-Investigator and must be submitted to the appropriate Independent Ethics Committees (IECs) for information and approval, in accordance with local requirements. Approval by IECs must be received before any changes can be implemented, except for changes necessary to eliminate an immediate hazard to study participants, or when the change involves only logistical or administrative aspects of the study.

10.2 Project management

The SaDAPT trial will be overseen and managed by a steering committee led by the Sponsor-Investigator Niklaus Labhardt. The steering committee will consist of representatives of the following project partners: Clinical Research Unit of the Swiss TPH, University Hospital Basel, Helse Nord Tuberculosis Initiative Malawi, Kamuzu University of Health Sciences (KUHES), SolidarMed Lesotho, Ministry of Health of Lesotho, London School of Hygiene and Tropical Medicine, Malawi Liverpool Wellcome Trust Clinical Research Programme, and the Liverpool School of Tropical Medicine.

For implementation in Lesotho, a subcontract with the Lesotho office of SolidarMed has been signed. SolidarMed is a Swiss not-for-profit organization, working in Lesotho for over 50 years (www.solidarmed.ch). Of note, SolidarMed is a long-standing implementing partner of the Sponsor-Investigator, the SwissTPH and the Ministry of Health in Lesotho. SolidarMed has the responsibility to coordinate the trial in Lesotho. For implementation of the study, SolidarMed will provide infrastructure, such as cars, office space, study personnel, and logistic and administrative support. The person responsible for the project at SolidarMed is Irene Ayakaka, technical director of SolidarMed Lesotho and National PI of this trial, who has extensive experience in project planning and implementation in Sub-Saharan Africa.

For implementation in Malawi, a subcontract with the Kamuzu University of Health Sciences (KUHES) will be signed. KUHES has the responsibility to coordinate the trial in Malawi. For implementation of the study KUHES will provide infrastructure, study personnel, and administrative support. The person responsible for the project at KUHES is Marriot Nliwasa, Research Group Leader and National PI of this trial, who has successfully led the implementation of various clinical trials on TB and HIV Malawi.

In each study country, the National PIs will support data collection and conduct data quality checks at national level. Data management at trial level will be hosted at SwissTPH under the responsibility of the trial statistician Tracy Glass who will also be responsible for statistical analyses of trial.

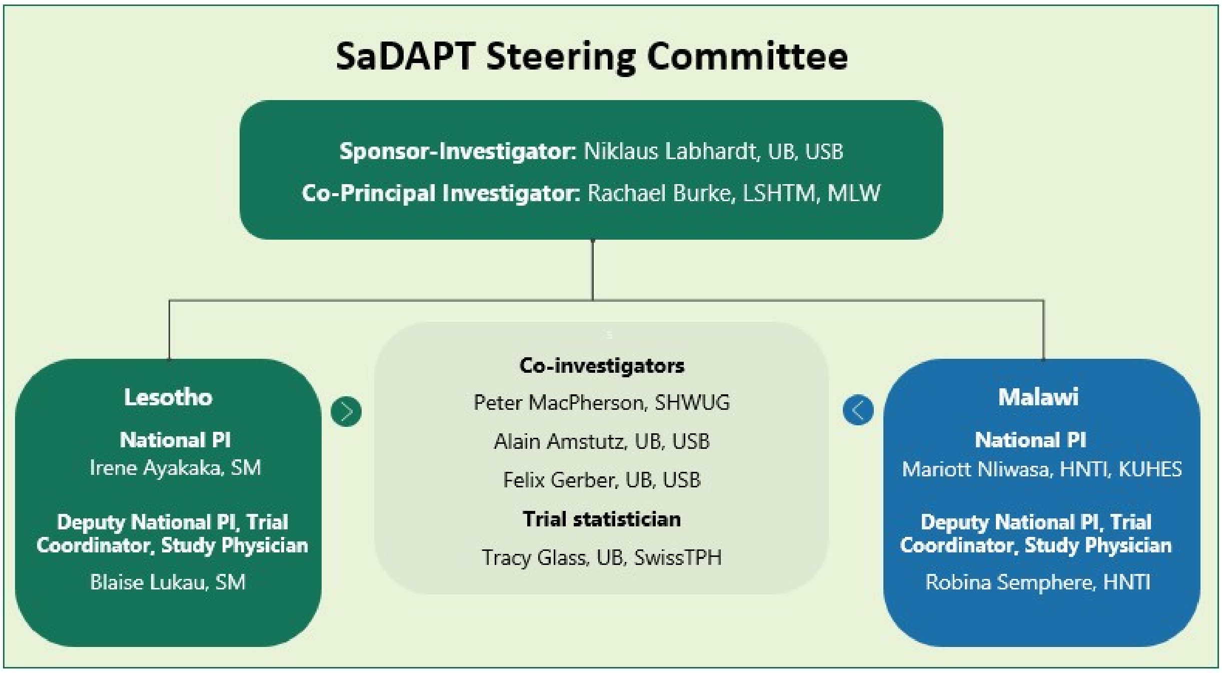

Figure 2 Organogram of the SaDAPT steering committee. *HNTI: Helse Nord Tuberculosis Initiative, KUHES: Kamuzu University of Health Sciences, LSHTM: London School of Hygiene and Tropical Medicine, LSTM: Liverpool School of Tropical Medicine, MLW: Malawi Liverpool Wellcome, SHWUG: School of Health and Wellbeing, University of Glasgow, SwissTPH: Swiss Tropical and Public Health Institute, UB: University of Basel, USB: University Hospital Basel, Switzerland*

11 ETHICAL CONSIDERATIONS

11.1 Independent Ethics Committee (IEC)

Ethics approval has been sought from the National Health Research and Ethics Committee of Lesotho, the College of Medicine Research and Ethics Committee (COMREC) of Malawi and the "Ethikkomission Nordwest- und Zentralschweiz" (EKNZ) in Switzerland prior to enrolment.

Any modifications to the protocol which may impact on the conduct of the study, potential benefit to the patient or patient safety, including changes of study objectives, study design, patient population, sample sizes, study procedures, or significant administrative aspects, will require a formal amendment to the protocol and will be submitted to the relevant IECs.

At the end of the trial, a written report will be submitted to all involved IECs.

11.2 Evaluation of the risk-benefit ratio

The group-specific timing for ART initiation relative to TB investigations has the following risks and benefits: In the "ART first" arm, the risk of IRIS is increased due to the earlier initiation of ART[11] whereas the risk of pre-ART attrition from care and thereby of AIDS-related complications, is minimized[20] and the opposite is true for the "TB results first" arm. For an in-depth risk-benefit assessment, we refer to the current WHO HIV guidelines[3] and the systematic review conducted to inform them on the question of when ART should be initiated in PLHIV with presumptive TB.[16] The rationale for the conduction of this trial is that based on the current evidence it remains unclear which of the strategies entails a more favorable overall risk-benefit profile.

11.3 Participant information and consent

Prior to enrolment, patients will asked for written informed consent (or assent with guardian consent in the case of adolescents with age 12 to 17 years) by trained site investigators.

Only individuals who are willing and able to consent will be recruited. We will not recruit incapacitated participants. We will recruit patients who are moderately unwell but retain capacity to consent to trial participation and whose medical care will be managed at primary care (i.e. not requiring transfer to higher level healthcare facility). All study staff taking consent will receive training in GCP and informed consent. Written information will be provided in English and Chichewa (Malawi) or Sesotho (Lesotho) and trained study staff will discuss the trial and answer questions in English, Chichewa or Sesotho. For participants who are illiterate, study staff will orally explain the participant information leaflet to the potential participant in detail in presence of an impartial witness. Illiterate participants will confirm informed consent with a thumbprint, countersigned by an impartial witness. No proxy consent for adults will be sought.

We will also include adolescents (age 12 to 17 years) in the trial. All study staff will receive training in working with and consenting adolescents. We will only recruit adolescents who are both willing and able to assent and who have a guardian (over 18 years) present who is willing and able to provide informed consent. We will not recruit incapacitated adolescents or adolescents who attend without a guardian. Written information to the adult guardian and the adolescent participant will be provided in English, Chichewa or Sesotho and be explained orally in detail to illiterate individuals in presence of an impartial witness. As above, guardian consent and adolescent assent will either be written with a signature, or a thumbprint countersigned by an impartial witness.

All potential participants will receive HIV and TB care from the clinic, according to national guidelines and supervised by national ART and TB treatment providers, irrespective of participation in the trial. Participants are free to withdraw their consent at any time and will continue to receive care as usual.

11.4 Registration of clinical trial

The trial has been registered at ClinicalTrials.gov (NCT05452616) and a summary of the protocol will be published in a peer-reviewed journal.

11.5 Participant confidentiality

The investigators will ensure that the participants' confidentiality will be maintained at all times during and after the trial, following procedures outlined in section 6.1 (enrolment procedures) and section 8 (data management).

11.6 Participants requiring particular protection

We will include adolescents aged 12 to 17 years in the trial if guardian consent is provided. Rationale for this and management of special protection is outlined in section 3.2. Compared to other age groups, adolescents have unfavorable HIV outcomes with increased attrition from care at all steps of the care cascade.[57] They might thus particularly benefit from access to uncomplicated, rapid initiation of ART. There exist no indications that one of the two approaches towards ART initiation applied in this trial might lead to unfavorable outcomes in adolescents.

11.7 Damage coverage

In the event of study-related damage or injuries, the liability of the trial insurance of the University Hopsital Basel provides compensation, except for claims that arise from participants' misconduct or gross negligence.

11.8 Participant compensation

Participants will participate in the trial in the course of their usual clinic visits, so should incur no extra costs related to transport and a minimal time burden. DBS collection at baseline may cause minimal discomfort.

In Lesotho, no compensation will be paid to participants. In Malawi, $10 equivalent in Malawi kwacha will be paid to participants.

12 QUALITY CONTROL AND QUALITY ASSURANCE: DESCRIPTION OF MEASURES

12.1 Risk management and possible constraints

12.1.1 Risks to viability of the study

We do not anticipate any serious threats to the study. Potential issues that we will navigate is the COVID-19 pandemic, which may reduce the number of people presenting to clinics and/or lead to change in dispensing and documentation practices. Another issue is lower than anticipated recruitment. If sample size is not reached after the anticipated 12 months recruitment period, we foresee a prolongation of recruitment at sites to be defined.

12.1.2 Risks and benefits for participants

Risks for participants are relatively small. Based on existing evidence, there is equipoise between the two interventions, as outlined in section 2 (background), section 0 (safety) and section 11 (ethics).

Apart from the group-specific timing of ART initiation, participation in the SaDAPT trial will not influence the care that participants receive at the study sites by routine clinical staff. Participants will be encouraged to return to the study clinic any time during the follow-up period to have medical complaints investigated and to receive care, as per local standards. The tracing of participants to ascertain the study endpoint between week 31 and 40 will be used to re-link participants to care and will thus help to minimize the number of participants lost to potentially life-saving HIV care.

The evidence that we aim to generate with this trial may help to establish the optimal approach to ART initiation in this large subgroup of PLHIV and has thus the potential to significantly improve health outcomes of these patients in the future.

12.2 Monitoring and Auditing

The Swiss TPH's Clinical Operations Unit (COU) will be responsible for developing the monitoring strategy. A monitoring plan will be developed before the study start. The monitoring will entail a systematic examination of study related activities and documents. Onsite monitoring in Lesotho will be conducted by a local monitoring team under the supervision of the COU. Onsite monitoring in Malawi will be conducted by the Malawi Liverpool Wellcome Clinical Research Support Unit (CRSU) in accordance with the monitoring plan provided by COU.

At least one monitoring visit at selected sites in both study countries will assess whether the study activities were conducted, and data were recorded, analyzed and accurately reported according to the approved protocol, GCP, and the applicable regulatory requirements.

The study documentation and the source data will be accessible to the monitoring team, the IECs and regulatory authorities upon request. The investigators will assist the inspectors in their duties, if requested.

All involved parties must keep the participant data strictly confidential.

12.3 Translations - Reference language

The reference language of the study documents is English, official language in both study countries. Whereas in both countries, healthcare professionals and thus all study staff is fluent in written and spoken English, this may not be the case for all participants. Therefore, the informed consent forms (ICFs) will be translated from an English master document into the local languages Sesotho (for Lesotho) and Chichewa (for Malawi) while all other study documents will be available in English exclusively. The quality of translation of the ICFs will be ensured by back-translation as well as by quality and comprehension checks by bilingual individuals not involved in the translation.

12.4 Storage of biological material and related health data

We will collect plasma and DBS samples for storage and subsequent analysis including HIV genotyping, resistance testing or other related research purposes. The samples collected in Lesotho will be stored for a maximum of five years at the laboratory of Seboche Hospital. All samples collected in Lesotho fall under the biobank agreement ("Biobanking regulations, v2.0") approved by the ethics committees in Lesotho and Switzerland.

In Malawi samples will be held for up to five years at the TB laboratory Kamuzu University of Health Sciences for studies related to same day ART or TB screening. Samples will remain the property of the government of Malawi. Participants will be asked to consent to samples being transferred out of Malawi for tests not available in Malawi. For instance, we plan to test for HIV genetic resistance – we are hopeful that by the end of the study this will be available in Malawi. However, should this not be the case we will apply for a Material Transfer Agreement (MTA) for shipping.

12.5 Project timeline

Quarter of year	***2022***				***2023***				***2024***			
Quarter of year	***1***	***2***	***3***	***4***	***1***	***2***	***3***	***4***	***1***	***2***	***3***	***4***
Preparation of study protocol, agreement, ethics approval, briefing of stakeholders, logistics preparation, recruitment of staff	x	x	x									
Recruitment of participants				x	x	x	x	x				
Follow-up period including tracing				x	x	x	x	x	x	x	x	x
Primary endpoint assessment					x	x	x	x	x	x	x	x
Data analysis, writing of publication										x	x	x

13 Funding

This trial is funded by a grant from the Swiss National Science Foundation (Project Number 32003B_205053/1), obtained by the Sponsor-Investigator Niklaus Labhardt. We attest that there is no conflict of interest and that this study will be conducted independently of outside influences in terms of specific intellectual, financial, and proprietary agendas. The funding source will have no role in the design of the trial, and will not be involved in data collection, data analysis, interpretation of the results, and writing of the manuscript.

14 Dissemination of results and publication policy

14.1 Dissemination to scientific community; including lead in publications

International scientific conferences and publications in scientific peer-reviewed journals will serve for wider dissemination of results. The requirements of the CONSORT statement[46] will be fulfilled. The study has been registered on ClinicalTrials.gov (NCT05452616) prior to the start of the trial and a summary of the study protocol will be published in a peer-reviewed journal. The current version of the International Committee of Medical Journal Editors (ICMJE) recommendations is applicable regarding authorship eligibility.[59] The use of professional writers is not intended.

An anonymized dataset of the study will be deposited at a public data repository once main-findings have been published.

14.2 Information of community and policy makers

Results of this study will be shared with stakeholders at district and national level in both study countries. In Lesotho, health care workers and stakeholders will be informed about the findings during district meetings headed by the District Health Management Team (DHMT) and at national level, the national research symposium of the Ministry of Health will serve as a platform to share the results and discuss their implications among the policy makers. In Malawi, results will be shared with the Blantyre District Health Office, the Department of HIV/AIDS and relevant HIV providing partners.

We will also organise results dissemination events in participating health facilities for staff and patients to hear the results of the study they participated in.

15 ART INITIATION COHORT SUB-STUDY

In parallel to the SaDAPT trial, we will observationally follow-up all PLHIV (re)initiating ART at the study sites in a prospective ART initiation cohort, independent of whether they are eligible for participation in the SaDAPT trial.

15.1 Rationale

The SaDAPT trial enrolls PLHIV (re)initiating ART who present with symptoms of a presumptive TB infection according to W4SS.[4] However, the sensitivity and specificity of the W4SS for TB case finding in PLHIV is limited and a considerable proportion of PLHIV are known to have subclinical TB disease before initiating ART.[4,60,61] It is unclear how relevant presence or absence of TB symptoms are for clinical management and outcomes, including occurrence of active TB.[62] Enrolling all PLHIV (re)initiating ART at the study facilities into an observational cohort in parallel to the recruitment for the SaDAPT trial shall allow to understand if and if yes to what extent characteristics and outcomes among PLHIV with and without TB symptoms who are (re)initiating ART differ and thus also help to weigh external validity and generalizability of the SaDAPT trial results.

15.2 Objectives

The objectives of the ART initiation cohort are to provide a description of baseline characteristics and outcomes of PLHIV (re)initiating ART in primary and secondary health facilities in Blantyre district, Malawi and Lesotho and to allow for an informal comparison between PLHIV (re)initiating ART with and without presumptive TB according to the W4SS. A further objective is to explore and describe the diagnostics, treatment and outcome of PLHIV with presumptive TB not eligible for participation in the SaDAPT RCT (i.e. due to reported pregnancy, signs of CNS disease or positive CrAg test) in routine care in primary and secondary health facilities in Blantyre district, Malawi and Lesotho. The ART initiation cohort will provide insights into the current management and outcomes of PLHIV (re)initiating ART in Malawi and Lesotho beyond the narrow question about timing of ART initiation in presumptive TB addressed with the SaDAPT RCT. The ART initiation cohort's objectives are thus not directly linked to the SaDAPT RCT's objectives, but add to them.

15.3 Study design and duration

The ART initiation cohort is an observational, prospective cohort study. Enrolment period for the ART initiation cohort is identical to the one for SaDAPT RCT, thus starting in October 2022 and ending when sample size for the SaDAPT RCT has been reached approximately 18 months later. Follow-up for the ART initiation cohort is maximum 24 months.

15.4 Study population and rationale

15.4.1 Inclusion and exclusion criteria

PLHIV (re)initiating ART at one of the study facilities during the recruitment period of the SaDAPT RCT meeting the following criteria will be included in the ART initiation cohort:

Inclusion criteria

- 12 years or older
- HIV-positive
- Not taking ART (naïve or reported no ART intake since 90 days or more)
- Planning to continue care at the study facility for at least 30 weeks
- Willing and able to consent (age 18 years or older) or assent with guardian consent (age 12-17 years)

Exclusion criteria

- Medical condition requiring referral to a higher level health facility at enrolment

The ART initiation cohort consists of consenting ART (re)initiators at the study sites, independent of whether they have presumptive TB or whether other exclusion criteria of the SaDAPT trial are present. Participants of the SaDAPT trial are part of the ART initiation cohort. The SaDAPT RCT ends after a follow-up period of maximum 40 weeks with the assessment of the trial's primary endpoint. After the termination of the follow-up of the randomized-controlled intervention, the participants of the SaDAPT RCT, will remain in care at the study facility and will be followed-up observationally like the other participants of the ART initiation cohort that were not part of the SaDAPT RCT.

15.4.2 Estimated sample size

No sample size calculations are performed. The enrolment will last until sample size for the SaDAPT RCT has been reached. We estimate the prevalence of any TB symptom among PLHIV (re)initiating ART between 30%-45%[36], thus sample size is estimated to reach between 1300 and 1800 participants.

15.5 Study procedures

Enrolment and follow-up of the ART initiation cohort will be performed by the study staff that is responsible for implementation of the SaDAPT trial, using the same data collection tool.

15.5.1 Screening and informed consent

The screening and informed consent process for the ART initiation cohort will follow the same principles as the processes for the SaDAPT trial and will be conducted by the same site investigators: Potential participants will be approached by a site investigator and asked to give verbal consent to study screening (equivalent to SaDAPT trial screening). As a result of the study screening, individuals will be classified either as not being eligible for any study, as being eligible for the ART initiation cohort only, but not for the SaDAPT trial or as being eligible for the SaDAPT trial (and therefore also for the ART initiation cohort).

Individuals meeting the criteria for participation in the SaDAPT trial, will be approached for informed consent for participation in the SaDAPT trial. The SaDAPT trial consent includes consent to further observational follow-up within the ART initiation cohort after termination of the SaDAPT follow-up period.

Individuals meeting the criteria for participation in the ART initiation cohort, but who do not meet the criteria for participation in the SaDAPT trial (for example due to absence of TB symptoms or due to a positive CrAg test) or who refuse participation in the SaDAPT trial, are being approached for consent to participate in the ART initiation cohort. As for the SaDAPT trial, the site investigator will discuss the cohort study with individuals identified as potential participants (and their guardian if age 12-17 years). An ART initiation cohort-specific information leaflet will be provided and all possible questions will be answered before the specific ICF may be signed. Consideration will be given to ensuring privacy and confidentiality. See section 11.3 for information about informed consent process, including for vulnerable participants as the same procedures apply for the ART initiation cohort

15.5.2 Baseline assessment

Enrolment will take place in an environment as private as possible, within the constraints of the clinic setting, so that confidentiality can be assured.

On enrolment, the following procedures will be performed:

- Brief clinical history and physical examination including:
    - HIV, ART and TB clinical history
    - Sociodemographic characteristics, health status, comorbidities and co-medications
    - Measurement of height and weight with determination of BMI and vital signs
    - Pregnancy status
    - Screening for signs of CNS disease

- Collection of DBS in Malawi and collection of plasma samples in Lesotho (DBS only if venous blood draw is not possible)

15.5.3 ART initiation

Routine staff will decide on timing of ART initiation depending on clinical situation and following national guidelines.[28,29] Study staff will document the date of ART initiation and relevant circumstances.

15.5.4 Follow-up

HIV care will be provided by routine clinic staff, according to national guidelines[28,29] with no intervening of study staff.

Study staff will document date of ART initiation, dates and results of possible TB investigations, dates of ART refills, dates and results of VLs and information about possible relevant complications based on data recorded by routine staff in the respective clinical registries. As for the SaDAPT trial, there will be register reviews 28 days and 30 weeks after enrolment to ensure complete and correct data collection. At the time of the 6-months routine VL measurement (window 22-30 weeks after enrolment), the same clinical assessment will be performed as for the SaDAPT trial participants and plasma samples (or DBS if blood draw not possible) will be collected at the study sites in Lesotho. At the study sites in Malawi, only for participants without a documented VL between 22 and 30 weeks after enrolment, a DBS might be collected as collection of plasma samples is not possible due to resource constraints. A subsample of ART initiation cohort participants will be traced back via phone calls and home visits to ascertain their outcome.

One year (56 weeks) and two years (112 weeks) after enrolment, register reviews will be conducted to assess the number of participants retained in care, the rate of viral suppression and the occurrence of SAEs and AESIs up to the respective time points. Again, a subsample of participants without documented VL result one and two years after enrolment will be traced back, if resources allow. In Lesotho plasma samples may be collected one and two years after enrolment if resources allow.

15.6 Safety considerations

The ART initiation cohort does not entail any intervention; all routine ART and TB services will remain as usual. Thus, no AE is likely to be related to study participation.

AEs will be solicited and recorded in the same way as for members of the SaDAPT trial. As all AEs are likely to be unrelated to study participation they do not need to be reported. Deaths should be reported as for the SaDAPT trial (section 7.1.6).

No monitoring, no DSMB and no formal interim analysis are planned for the ART initiation cohort.

15.7 Data management

For data management, data safety and confidentiality, the same procedures apply as for the SaDAPT trial (see section 8).

15.8 Statistics

Cohort baseline characteristics and outcomes will be presented descriptively, no formal statistical analysis will be performed.

15.9 Ethical considerations

15.9.1 Risk benefit assessment and compensation

The ART initiation cohort is purely observational. Risk from participation is minimal. Data confidentiality will be maintained. Participants may benefit from improved care through signposting of missing HIV and/or TB drug prescription for eligible participants to routine staff and through re-linkage to care during

the tracing of participants lost to follow-up. The evidence generated with this cohort study will contribute valuable evidence to the understanding of current management and outcomes of PLHIV (re)initiating ART in southern Africa. Participants in Malawi will receive a compensation of 1000 Malawi kwacha (circa 1.25 US dollar), no compensation will be paid to participants in Lesotho.

15.9.2 Rationale for inclusion of vulnerable subjects

We will include adolescents aged 12 to 17 years if assent and guardian consent is provided and consenting pregnant women in the ART initiation cohort. Both groups are often excluded from interventional studies due to safety or ethical concerns. Evidence on these subgroups to improve treatment approaches is required as for all other PLHIV. We will include them in this observational study, to generate some evidence on current management and outcomes of these specific subgroups of PLHIV without exposing them to the risk of an intervention.

16 SERUM PROTEOME SUB-STUDY: PREDICTION OF CINICAL PHENOTYPES IN PEOPLE LIVING WITH HIV USING THE SERUM PROTEOME

This is a laboratory-based sub-study that will be conducted using the plasma samples and clinical data collected as part of SaDAPT trial and ART initiation cohort at the study sites in Lesotho.

16.1 Background and rationale

Mycobacterium tuberculosis (MTB) infection is ancient: models suggest that MTB might have emerged as a human pathogen around 400,000 years ago [63]. Over this long period, MTB and humans have co-evolved to reach a balance; MTB infects many people—approximately 28% of the human population have been exposed to MTB —but over 90% of infected individuals do not develop disease[64]. HIV infection remains one of the most important risk factors for developing active TB[65].

Diagnosis of active TB remains a challenge: PLHIV typically present with disseminated disease delaying appropriate treatment[66]. An additional complication of HIV/TB co-infection is increased risk for IRIS after initiation of antiretroviral treatment. IRIS develops once HIV viremia is suppressed due to the reconstitution of the immune system. The recovering immune system over-reacts to ongoing infections (paradoxical IRIS) or recognizes infections which were previously not detected due the HIV induced immune suppression (unmasking IRIS). Predicting IRIS in PLHIV is impossible with current diagnostic methods.

In this sub-study, we will screen samples from the SaDAPT trial and the SaDAPT ART initiation cohort in Lesotho to map the landscape of circulating inflammatory markers using cutting the edge proteomics in PLHIV with high risk of active TB and IRIS. The proteome will be deconvoluted using computational biology with the aim to develop diagnostics for active TB disease and predictors for IRIS.

A test based on circulating proteins to predict IRIS has significant advantages: proteins are easy to measure in peripheral blood which allows the development of a point of care test in a straightforward way. In turn, a point of care test to predict IRIS would facilitate prevention and treatment of IRIS.

Today, IRIS recommendations are primarily based on disease entities. For example, in the case of TB meningitis and TB pericarditis, steroid treatment as prevention for IRIS is recommended. However, it is well known that lymph node TB is also prone to IRIS type reactions. However, for lymph node TB steroids are generally not recommended. A point of care test to predict IRIS would allow personalizing the clinical approach to IRIS prevention by going beyond arbitrary disease entities.

This sub-study has the potential to directly affect clinical care of a highly vulnerable population at high risk for unfavorable outcomes.

16.2 Objectives

- To diagnose active TB in PLHIV presenting with clinical symptoms
- To predict IRIS in PLHIV undergoing antiretroviral treatment
- To explore factors associated with control or progress of both TB and HIV infections

16.3 Approach

16.3.1 Sample collection and assessment of the clinical phenotype

For this sub-study, plasma samples from the SaDAPT trial and the ART initiation cohort collected at study sites in Lesotho will be used. For the assessment of the clinical phenotype (i.e. CD4 cell count, VL, occurrence of active TB or IRIS), the data collected during the SaDAPT trial and the ART initiation cohort as described above will be used. No clinical data or samples other than outlined in the protocol sections of the SaDAPT trial and the ART initiation cohort will be collected for this sub-study.

16.3.2 Measurement of the proteome

Proteome analysis will be conducted at the Department for Infectious Diseases and Hospital Epidemiology of the University Hospital Zurich in Switzerland under the supervision of Prof. Johannes Nemeth. Before shipment of the plasma samples to Switzerland, a specific Material Transfer Agreement will be submitted to NH-REC for review.

At the Department for Infectious Diseases and Hospital Epidemiology laboratory, plasma samples will be processed using the PreOmics iST kit in a 96 well format. Quantitative data will be acquired in diaPASEF mode (data independent acquisition – parallel accumulation serial fragmentation) on a timsTOF Pro mass spectrometer equipped with nanoElute chromatography system (Bruker)[66]. This method uses trapped ion mobility separations to increase sensitivity and resolving power combined with data independent acquisition to ensure robustness and data completeness. Preliminary data from serum samples shows we can robustly quantify 350-400 serum proteins from two microliters of human serum at a throughput of 50 samples per day making this compatible with analysis of large-scale cohorts. Importantly, only proteome analysis and no human genome analyses will be conducted[67].

16.3.3 Deconvolution of proteomic maps using computational biology

This study requires deconvolution of high dimensional data (the proteome) into a clinically meaningful predictor. We will use advanced statistical methods to deconvolute the high dimensional data. Specifically, we will primarily focus on the development of a proteome based voting score, given our publishing record and our experience with the diagnostic score development based on the published pairwise support vector machine (SVM) parameters and the robustness of the approach[68]. However, we will also employ random forest models and deep neuronal network approaches and compare the diagnostic accuracy of different techniques.

We will follow a training – test cohort strategy whereby approximately 2/3 of all samples will be used as input into the machine learning algorithms. The remaining 1/3 of samples will be used as test set to evaluate the performance of the training set. Sample allocation to test and training sets is randomized. For external validation, we will test our proteome signature against already published proteome signatures in patients with active TB and HIV infection.

16.4 Ethical considerations

16.4.1 Risk benefit assessment

No risk for participants is associated with the proteome sub-study as it only entails laboratory assessments of samples collected during the SaDAPT trial/ART initiation cohort and linking it to anonymized clinical data collected as part of the SaDAPT trial/ART initiation cohort. Samples will be labelled with the participant number while the participant identification log will remain with the site investigators during the study and at the SolidarMed archive in Lesotho after the termination of the study activities at the sites, thus all assessments for the proteome sub-study will be fully anonymous. No human genome analysis will be performed.

Predicting active TB in high-risk patients such as PLHIV remains woefully inadequate. Current tests (interferon release assays, transcriptional signatures) are complicated to perform, expensive and display underwhelming diagnostic accuracy. A protein based diagnostic signature for prediction of TB and/or IRIS paves the way for a simple, cheap point of care test.

17 References

1 UNAIDS. Global HIV & AIDS statistics – 2021 fact sheet. 2021. https://www.unaids.org/sites/default/files/media_asset/UNAIDS_FactSheet_en.pdf (accessed Oct 18, 2021).

2 World Health Organization. Global tuberculosis report 2021. Geneva: World Health Organization, 2021 https://apps.who.int/iris/handle/10665/346387 (accessed Oct 25, 2021).

3 World Health Organization. Consolidated guidelines on HIV prevention, testing, treatment, service delivery and monitoring: recommendations for a public health approach, 2021 update. Geneva: World Health Organization, 2021 https://apps.who.int/iris/handle/10665/342899 (accessed Oct 11, 2021).

4 Hamada Y, Lujan J, Schenkel K, Ford N, Getahun H. Sensitivity and specificity of WHO's recommended four-symptom screening rule for tuberculosis in people living with HIV: a systematic review and meta-analysis. *Lancet HIV* 2018; **5**: e515–23.

5 Getahun H, Kittikraisak W, Heilig CM, *et al.* Development of a standardized screening rule for tuberculosis in people living with HIV in resource-constrained settings: individual participant data meta-analysis of observational studies. *PLoS Med* 2011; **8**: e1000391.

6 Dhana A, Hamada Y, Kengne AP, *et al.* Tuberculosis screening among ambulatory people living with HIV: a systematic review and individual participant data meta-analysis. *Lancet Infect Dis* 2021; : S147330992100387X.

7 Organization WH. Guidelines: updated recommendations on HIV prevention, infant diagnosis, antiretroviral initiation and monitoring. World Health Organization, 2021 https://apps.who.int/iris/handle/10665/340190 (accessed April 15, 2021).

8 Meintjes G, Lawn SD, Scano F, *et al.* Tuberculosis-associated immune reconstitution inflammatory syndrome: case definitions for use in resource-limited settings. *Lancet Infect Dis* 2008; **8**: 516–23.

9 Dhasmana DJ, Dheda K, Ravn P, Wilkinson RJ, Meintjes G. Immune reconstitution inflammatory syndrome in HIV-infected patients receiving antiretroviral therapy : pathogenesis, clinical manifestations and management. *Drugs* 2008; **68**: 191–208.

10 Church LWP, Chopra A, Judson MA. Paradoxical Reactions and the Immune Reconstitution Inflammatory Syndrome. 2017; **5**. DOI:doi:10.1128/microbiolspec.TNMI7-0033-2016.

11 Abay SM, Deribe K, Reda AA, *et al.* The Effect of Early Initiation of Antiretroviral Therapy in TB/HIV-Coinfected Patients: A Systematic Review and Meta-Analysis. *J Int Assoc Provid AIDS Care* 2015; **14**: 560–70.

12 Walker NF, Stek C, Wasserman S, Wilkinson RJ, Meintjes G. The tuberculosis-associated immune reconstitution inflammatory syndrome: recent advances in clinical and pathogenesis research. *Curr Opin HIV AIDS* 2018; **13**: 512–21.

13 Namale PE, Abdullahi LH, Fine S, Kamkuemah M, Wilkinson RJ, Meintjes G. Paradoxical TB-IRIS in HIV-infected adults: a systematic review and meta-analysis. *Future Microbiol* 2015; **10**: 1077–99.

14 World Health Organization. Guidelines: updated recommendations on HIV prevention, infant diagnosis, antiretroviral initiation and monitoring. Geneva: World Health Organization, 2021 https://apps.who.int/iris/handle/10665/340190 (accessed Nov 17, 2021).

15 World Health Organization. Guidelines for managing advanced HIV disease and rapid initiation of antiretroviral therapy, July 2017. Geneva: World Health Organization, 2017 https://apps.who.int/iris/handle/10665/255884 (accessed Oct 25, 2021).

16 Burke RM, Rickman HM, Singh V, *et al.* Same-day antiretroviral therapy initiation for people living with HIV who have tuberculosis symptoms: a systematic review. *HIV Med* 2021; : hiv.13169.

17 Joint United Nations Programme on HIV/AIDS. UNAIDS Data 2021. 2021. https://www.unaids.org/en/resources/documents/2021/2021_unaids_data (accessed Dec 14, 2021).

18 Rosen S, Maskew M, Fox MP, *et al.* Initiating Antiretroviral Therapy for HIV at a Patient's First Clinic Visit: The RapIT Randomized Controlled Trial. *PLoS Med* 2016; **13**: e1002015.

19 Lilian RR, Rees K, McIntyre JA, Struthers HE, Peters RPH. Same-day antiretroviral therapy initiation for HIV-infected adults in South Africa: Analysis of routine data. *PLOS ONE* 2020; **15**: e0227572.

20 Labhardt ND, Ringera I, Lejone TI, *et al.* Effect of Offering Same-Day ART vs Usual Health Facility Referral During Home-Based HIV Testing on Linkage to Care and Viral Suppression Among Adults With HIV in Lesotho: The CASCADE Randomized Clinical Trial. *Jama* 2018; **319**: 1103–12.

21 Koenig SP, Dorvil N, Dévieux JG, *et al.* Same-day HIV testing with initiation of antiretroviral therapy versus standard care for persons living with HIV: A randomized unblinded trial. *PLOS Med* 2017; **14**: e1002357.

22 Pilcher CD, Ospina-Norvell C, Dasgupta A, *et al.* The Effect of Same-Day Observed Initiation of Antiretroviral Therapy on HIV Viral Load and Treatment Outcomes in a US Public Health Setting. *J Acquir Immune Defic Syndr* 2017; **74**: 44–51.

23 Cohen MS, Chen YQ, McCauley M, *et al.* Prevention of HIV-1 Infection with Early Antiretroviral Therapy. 2011; **365**: 493–505.

24 Panel on Antiretroviral Guidelines for Adults and Adolescents. Guidelines for the Use of Antiretroviral Agents in Adults and Adolescents with HIV. Department of Health and Human Services. Available at https://clinicalinfo.hiv.gov/sites/default/files/guidelines/documents/AdultandAdolescentGL.pdf. Accessed 10/25/2021. .

25 Government of Lesotho Ministry of Health Lesotho. Approach to the Management of Advanced HIV Disease (AHD). 2020.

26 Republic of South Africa National Department of Health. 2019 ART Clinical Guidelinesfor the Management of HIV in Adults, Pregnancy, Adolescents, Children, Infants and Neonates. 2020 https://www.knowledgehub.org.za/system/files/elibdownloads/2020-05/2019%20ART%20Guideline%2028042020%20pdf.pdf (accessed Oct 25, 2021).

27 Ryom L, Cotter A, De Miguel R, *et al.* 2019 update of the European AIDS Clinical Society Guidelines for treatment of people living with HIV version 10.0. *HIV Med* 2020; **21**: 617–24.

28 Ministry of Health and Population M. 4th Edition of The Malawi Guidelines for Clinical Management of HIV in Children and Adults. 2018.

29 Ministry of Health Lesotho. National guidelines on the use of antiretroviral therapy for HIV prevention and treatment, Sixth edition. 2022; published online Jan.

30 Török ME, Yen NT, Chau TT, *et al.* Timing of initiation of antiretroviral therapy in human immunodeficiency virus (HIV)--associated tuberculous meningitis. *Clin Infect Dis* 2011; **52**: 1374–83.

31 Bahr N, Boulware DR, Marais S, Scriven J, Wilkinson RJ, Meintjes G. Central nervous system immune reconstitution inflammatory syndrome. *Curr Infect Rep* 2013; **15**: 583–93.

32 Eshun-Wilson I, Okwen MP, Richardson M, Bicanic T. Early versus delayed antiretroviral treatment in HIV-positive people with cryptococcal meningitis. *Cochrane Database Syst Rev* 2018; **7**: Cd009012.

33 Havlir DV, Kendall MA, Ive P, *et al.* Timing of antiretroviral therapy for HIV-1 infection and tuberculosis. *N Engl J Med* 2011; **365**: 1482–91.

34 Abdool Karim SS, Naidoo K, Grobler A, *et al.* Timing of initiation of antiretroviral drugs during tuberculosis therapy. *N Engl J Med* 2010; **362**: 697–706.

35 Uthman OA, Okwundu C, Gbenga K, *et al.* Optimal Timing of Antiretroviral Therapy Initiation for HIV-Infected Adults With Newly Diagnosed Pulmonary Tuberculosis: A Systematic Review and Meta-analysis. *Ann Intern Med* 2015; **163**: 32–9.

36 Brennan A, Maskew M, Larson BA, *et al.* Prevalence of TB symptoms, diagnosis and treatment among people living with HIV (PLHIV) not on ART presenting at outpatient clinics in South Africa and Kenya: baseline results from a clinical trial. *BMJ Open* 2020; **10**: e035794.

37 Rosen S, Maskew M, Larson BA, *et al.* Simplified clinical algorithm for identifying patients eligible for same-day HIV treatment initiation (SLATE): Results from an individually randomized trial in South Africa and Kenya. *PLoS Med* 2019; **16**: e1002912.

38 Maskew M, Brennan AT, Fox MP, *et al.* A clinical algorithm for same-day HIV treatment initiation in settings with high TB symptom prevalence in South Africa: The SLATE II individually randomized clinical trial. *PLoS Med* 2020; **17**: e1003226.

39 Lawn SD, Campbell L, Kaplan R, Little F, Morrow C, Wood R. Delays in starting antiretroviral therapy in patients with HIV-associated tuberculosis accessing non-integrated clinical services in a South African township. *BMC Infect Dis* 2011; **11**: 258.

40 Fox MP, Rosen S, Geldsetzer P, Bärnighausen T, Negussie E, Beanland R. Interventions to improve the rate or timing of initiation of antiretroviral therapy for HIV in sub-Saharan Africa: meta-analyses of effectiveness. *J Int AIDS Soc* 2016; **19**: 20888.

41 Rosen S, Fox MP, Larson BA, *et al.* Accelerating the Uptake and Timing of Antiretroviral Therapy Initiation in Sub-Saharan Africa: An Operations Research Agenda. *PLoS Med* 2016; **13**: e1002106.

42 Joint United Nations Programme on HIV/AIDS (UNAIDS). Young people and HIV UNAIDS 2021. 2021.

43 Bowen L, Nath A, Smith B. CNS immune reconstitution inflammatory syndrome. *Handb Clin Neurol* 2018; **152**: 167–76.

44 Amstutz A, Nsakala BL, Vanobberghen F, *et al.* Switch to second-line versus continued first-line antiretroviral therapy for patients with low-level HIV-1 viremia: An open-label randomized controlled trial in Lesotho. *PLOS Med* 2020; **17**: e1003325.

45 Eaton JW, Dwyer-Lindgren L, Gutreuter S, *et al.* Naomi: a new modelling tool for estimating HIV epidemic indicators at the district level in sub-Saharan Africa. *J Int AIDS Soc* 2021; **24**. DOI:10.1002/jia2.25788.

46 National Tuberculosis Control Programme (Malawi) C for SRU of MWHO (WHO), C for DC and P (CDC). Malawi Tuberculosis Prevalence Survey 2013-2014. 2015.

47 MacPherson P, Webb EL, Kamchedzera W, *et al.* Computer-aided X-ray screening for tuberculosis and HIV testing among adults with cough in Malawi (the PROSPECT study): A randomised trial and cost-effectiveness analysis. *PLOS Med* 2021; **18**: e1003752.

48 Divala TH, Fielding KL, Sloan DJ, *et al.* Accuracy and consequences of using trial-of-antibiotics for TB diagnosis (ACT-TB study): protocol for a randomised controlled clinical trial. *BMJ Open* 2020; **10**: e033999.

49 Ministry of Health Lesotho. Kingdom of Lesotho National Tuberculosis Programme Policy and Manual. 2015.

50 Ministry of Health Malawi. Malawi National Tuberculosis Programme Manual, Seventh Edition. 2012.

51 Harris PA, Taylor R, Minor BL, *et al.* The REDCap consortium: Building an international community of software platform partners. *J Biomed Inform* 2019; **95**: 103208–103208.

52 Schulz KF, Altman DG, Moher D. CONSORT 2010 statement: Updated guidelines for reporting parallel group randomised trials. 2010; **1**: 8.

53 Piaggio G, Elbourne DR, Pocock SJ, Evans SJW, Altman DG. Reporting of Noninferiority and Equivalence Randomized Trials. 2010; : 11.

54 Kahan BC, Morris TP. Reporting and analysis of trials using stratified randomisation in leading medical journals: review and reanalysis. *BMJ* 2012; **345**: e5840–e5840.

55 Pocock SJ, Assmann SE, Enos LE, Kasten LE. Subgroup analysis, covariate adjustment and baseline comparisons in clinical trial reporting: current practiceand problems. *Stat Med* 2002; **21**: 2917–30.

56 Points to consider on switching between superiority and non-inferiority. *Br J Clin Pharmacol* 2001; **52**: 223–8.

57 Casale M, Carlqvist A, Cluver L. Recent Interventions to Improve Retention in HIV Care and Adherence to Antiretroviral Treatment Among Adolescents and Youth: A Systematic Review. *AIDS Patient Care STDs* 2019; **33**: 237–52.

58 Campbell MK, Piaggio G, Elbourne DR, Altman DG, for the CONSORT Group. Consort 2010 statement: extension to cluster randomised trials. *BMJ* 2012; **345**: e5661–e5661.

59 International Committee of Medical Journal Editors. Recommendations for the Conduct, Reporting, Editing and Publication of Scholarly Work in Medical Journals. http://www.ICMJE.org (accessed Dec 19, 2021).

60 Bajema KL, Bassett IV, Coleman SM, *et al.* Subclinical tuberculosis among adults with HIV: clinical features and outcomes in a South African cohort. *BMC Infect Dis* 2019; **19**: 14.

61 Modi S, Cavanaugh JS, Shiraishi RW, *et al.* Performance of Clinical Screening Algorithms for Tuberculosis Intensified Case Finding among People Living with HIV in Western Kenya. *PLOS ONE* 2016; **11**: e0167685.

62 Drain PK, Bajema KL, Dowdy D, *et al.* Incipient and Subclinical Tuberculosis: a Clinical Review of Early Stages and Progression of Infection. *Clin Microbiol Rev* 2018; **31**. DOI:10.1128/CMR.00021-18.

63 Chisholm RH, Trauer JM, Curnoe D, Tanaka MM. Controlled fire use in early humans might have triggered the evolutionary emergence of tuberculosis. *Proc Natl Acad Sci* 2016; **113**: 9051–6.

64 Houben RMGJ, Dodd PJ. The Global Burden of Latent Tuberculosis Infection: A Re-estimation Using Mathematical Modelling. *PLOS Med* 2016; **13**: e1002152.

65 Kwan CK, Ernst JD. HIV and Tuberculosis: a Deadly Human Syndemic. *Clin Microbiol Rev* 2011; **24**: 351–76.

66 Kulak NA, Pichler G, Paron I, Nagaraj N, Mann M. Minimal, encapsulated proteomic-sample processing applied to copy-number estimation in eukaryotic cells. *Nat Methods* 2014; **11**: 319–24.

67 Nemeth J, Vongrad V, Metzner KJ, *et al.* In Vivo and in Vitro Proteome Analysis of Human Immunodeficiency Virus (HIV)-1-infected, Human CD4+ T Cells. *Mol Cell Proteomics* 2017; **16**: S108–23.

68 Duffy FJ, Olson GS, Gold ES, *et al.* Use of a Contained *Mycobacterium tuberculosis* Mouse Infection Model to Predict Active Disease and Containment in Humans. *J Infect Dis* 2022; **225**: 1832–40.
